# Integrated Exposure–Response of Dupilumab in Children, Adolescents, and Adults With Atopic Dermatitis Using Categorical and Continuous Efficacy Assessments: A Population Analysis

**DOI:** 10.1007/s11095-023-03616-8

**Published:** 2023-12-11

**Authors:** Emily Briggs, Mohamed A. Kamal, Matthew P. Kosloski, Ian Linsmeier, Natalie Jusko, Nancy Dolphin, Jason Chittenden, Eric L. Simpson, Amy S. Paller, Elaine C. Siegfried, Brad Shumel, Noah A. Levit, Ashish Bansal, John D. Davis, Sunny Chapel, David E. Smith, Nidal Huniti

**Affiliations:** 1 Department of Pharmaceutical Sciences, College of Pharmacy, University of Michigan, Ann Arbor, MI USA; 2 A2-Ai, Ann Arbor, MI USA; 3 Regeneron Pharmaceuticals Inc, Tarrytown, NY USA; 4 Arcus Biosciences, Hayward, CA USA; 5 Amador Bioscience, Ann Arbor, MI USA; 6 Oregon Health and Science University, Portland, OR USA; 7 Northwestern University Feinberg School of Medicine, Chicago, IL USA; 8 Ann and Robert H. Lurie Children’s Hospital, Chicago, IL USA; 9 Saint Louis University, St. Louis, MO USA; 10 Cardinal Glennon Children’s Hospital, St. Louis, MO USA; 11 Dermatology Physicians of Connecticut, Fairfield, CT USA

**Keywords:** atopic dermatitis, dupilumab, exposure–response, pediatrics

## Abstract

**Background:**

While the majority of patients with atopic dermatitis (AD) achieve disease control with dupilumab treatment, there is variability in which patients achieve clear disease. The predictors of these responses are currently unclear. Integrated models were developed to evaluate the exposure–response (E-R) relationship of dupilumab in children, adolescents, and adults with AD.

**Methods:**

Data from six Phase II and III clinical studies were pooled (2,366 adults [> 18 years], 243 adolescents [≥ 12 to < 18 years] and 359 children [≥ 6 to < 12 years]) for model development. Efficacy was assessed using the Eczema Area and Severity Index (EASI) and Investigator’s Global Assessment (IGA). Indirect response models were applied to link measures of efficacy and functional serum dupilumab concentrations. The covariates on individual placebo-corrected response were assessed. Clinical trial scenarios were simulated to compare E-R relationships across age groups. Safety was not explored.

**Results:**

After correcting for differences in placebo response and dupilumab exposure: 1) older age, higher body weight, lower baseline thymus and activation-regulated chemokine, and Asian race were associated with slightly lower EASI response, and no clear covariates were identified on IGA response; 2) clinical trial simulations generally showed slightly higher response at a given dupilumab concentration in children compared to adults and adolescents with severe and moderate AD.

**Conclusions:**

The collectively tested covariates explain some of the variability in dupilumab response in patients with AD. Patients in all age groups showed adequate response to dupilumab; however, children showed slightly higher drug effects compared to adults and adolescents at equivalent concentrations.

**Supplementary Information:**

The online version contains supplementary material available at 10.1007/s11095-023-03616-8.

## Introduction

Atopic dermatitis (AD) is a chronic skin disease characterized by inflammation and pruritus. According to the Global Burden of Disease (GBD) study from 2017, AD has the highest burden of all skin diseases, and is the most common pediatric inflammatory skin disease, affecting around 20% of children in high-income countries [1]. Moreover, AD is a heterogeneous disease in both time of onset, severity, and duration, with most patients presenting with symptoms early in life, while some develop symptoms late into adulthood (late-onset). AD has a chronic impact on all patients, with some patients experiencing lifelong disease while others may experience a more dynamic clinical course with shifting severity and sometimes disease remittance by adolescence or early adulthood [1].

Current standard of care for patients with AD includes use of topical agents such as topical corticosteroids (TCS), calcineurin inhibitors, PDE-4 inhibitors, and Janus kinase inhibitors, and phototherapy [2–5]. In patients with moderate-to-severe disease, dupilumab is indicated for patients aged 6 months and above, with or without concomitant use of TCS, with approval having been extended down to patients aged 6 months to 5 years in June 2022 [6, 7]. Dupilumab is a fully human VelocImmune^®^-derived [8, 9] monoclonal antibody that blocks the shared receptor component for interleukin (IL)-4 and IL-13, thus inhibiting signaling of both IL-4 and IL-13 [10]. Dupilumab has been shown to be effective in treating moderate-to-severe AD in clinical trials in adult (≥ 18 years), adolescent (12 to 17 years), and pediatric (6 to 12 years, and 6 months to 5 years) populations [11–15], as well as several other type 2 inflammatory disorders, including asthma [16–18], chronic rhinosinusitis with nasal polyposis (CRSwNP) [19], eosinophilic esophagitis [20], and prurigo nodularis [21].

While dupilumab has demonstrated efficacy in the treatment of AD over a wide range of patient ages, variations in the design of clinical trials across evaluated age groups [11–15] have made it difficult to directly assess any differences in drug response among pediatric and adult patients based on observed data. For example, while weight-tiered dose regimens were selected in pediatric patients to closely match drug concentrations in adults, steady-state trough concentrations of dupilumab in adolescents on average are slightly lower compared to adults, while those in children ≥ 6 years to < 12 years of age are slightly higher [22]. Moreover, clinical trials have shown different placebo responses in adults, adolescents, and children [11–15]. Furthermore, adult and pediatric trials differed with respect to baseline AD disease severity and co-administration (or lack) of TCS therapy. All these factors may confound a comparative exposure–response (E-R) analysis of dupilumab between adult and pediatric patients with AD.

To date, there has not been a comprehensive E-R analysis of dupilumab in adults and pediatric patients with AD assessing the entire time-course of drug concentration and response, which adjusts for potential confounding factors and evaluates both continuous and categorical measures of efficacy. Previous analysis has been performed in pediatric populations [22] but a major limitation of the study was that it was performed sequentially, and only utilized data from Week 16 of the clinical trials, rather than the full time-course of drug concentrations and responses. It also only included populations for which clinical trial data were available, thereby not covering children with moderate AD or adolescents receiving concomitant TCS. In the current study, we performed non-linear mixed-effects modeling to characterize the full time-course E-R of dupilumab in adult and pediatric (≥ 6 to < 18 years of age) patients with moderate-to-severe AD. The E-R analysis integrated dose-ranging data from Phase II and III clinical trials in which patients received ≥ 16 weeks of treatment; efficacy assessments included the continuous Eczema Area and Severity Index (EASI) and the categorical Investigator’s Global Assessment (IGA). We aimed to 1) identify covariates explaining patient variability in drug response; and 2) explore differences in dupilumab E-R between adult and pediatric patients after adjusting for potential confounding factors.

## Materials and Methods

### Study Participants

Data from one Phase IIb and five Phase III clinical studies were pooled to support the population E-R analyses, including one study in children (6–11 years of age) with severe AD (NCT03345914) [11], one study in adolescents (12–17 years of age) with moderate or severe AD (NCT03054428) [12], and four studies in adults with moderate or severe AD (NCT01859988 [13], NCT02277769 [14], NCT02277743 [14], NCT02260986 [15]). Individual study designs and clinical trial identifiers are listed in Table I. Across all studies and age groups, dupilumab was administered subcutaneously, with a single loading dose administered on Day 1 equivalent to twice the maintenance dose, either alone (NCT03054428, NCT01859988, NCT02277769, and NCT02277743) or with concomitant TCS (TNCT03345914 and NCT02260986). Study protocols were approved by medical ethics committees and institutional review boards of the participating centers. All patients or their caregivers provided written informed consent before enrollment.

**Table I:** Summary of studies included in the population modeling analysis

Study ID	Phase	Patient age category (age range, years)	AD severity (IGA score)	± TCS	Dosage/Drug regimen^a^	No. patients (completed treatment/ planned)^b^	IGA and EASI score timepoints (study Week)
NCT03345914 R668-AD-1652 [11]	III	Children (6, 12)	Severe (IGA = 4)	+ TCS	Day 1 loading dose: 2 × maintenance dose • Dupilumab SC q2w + TCS: ◦ 100 mg for patients < 30 kg ◦ 200 mg for patients ≥ 30 kg • Dupilumab 300 mg SC q4w + TCS • Placebo SC q2w + TCS^d^	*N* = 367 Placebo: 114/123 Dupilumab: 237/244	Screening, baseline, Week 1, 2, 3, 4, 8, 12, EOT (Week 16), follow-up period (Week 20, 24), EOS (Week 28), unscheduled visit, early termination^e^
NCT03054428 R668-AD-1526 [12]	III	Adolescents (12, 18)	Moderate (IGA = 3) Severe (IGA = 4)	– TCS	Day 1 loading dose: 2 × maintenance dose • Dupilumab SC q2w: ◦ 200 mg for patients < 60 kg ◦ 300 mg for patients ≥ 60 kg • Dupilumab 300 mg SC q4w • Placebo SC q2w	*N* = 251 Placebo: 76/85 Dupilumab: 155/166	Screening, baseline, Week 1, 2, 3, 4, 8, 12, EOT (Week 16), follow-up period (Week 20, 24), EOS (Week 28), unscheduled visit, early termination
NCT01859988 R668-AD-1021 [13]	IIb	Adults (18, 75)	Moderate (IGA = 3) Severe (IGA = 4)	– TCS	Day 1 loading dose: 2 × maintenance dose (4 × for 100 mg SC q4w only) • Dupilumab ◦ 100 mg SC q4w ◦ 300 mg SC q4w ◦ 200 mg SC q2w ◦ 300 mg SC q2w ◦ 300 mg SC qw • Placebo SC qw	*N* = 380 Placebo: 53/61 Dupilumab: 294/319	Screening, baseline, Week 1, 2, 3, 4, 6, 8, 10, 12, 14, 15, EOT (Week 16), 18, 20, 22, 24, 26, 28, 30, EOS (Week 32), unscheduled visit, early termination
NCT02277769 R668-AD-1416 [14]^c^	III	Adults (18, 75)	Moderate (IGA = 3) Severe (IGA = 4)	– TCS	Day 1 loading dose: 2 × maintenance dose • Dupilumab 300 mg SC qw • Dupilumab 300 mg SC q2w • Placebo SC qw	*N* = 708 Placebo: 190/236 Dupilumab: 441/472	Screening, baseline, Week 1, 2, 4, 6, 8, 12, EOT (Week 16), follow-up period (Week 20, 24), EOS (Week 28), unscheduled visit, early termination^e^
NCT02277743 R668-AD-1334 [14]^c^	III	Adults (18, 75)	Moderate (IGA = 3) Severe (IGA = 4)	– TCS	Day 1 loading dose: 2 × maintenance dose • Dupilumab 300 mg SC qw • Dupilumab 300 mg SC q2w • Placebo SC qw	*N* = 671 Placebo: 184/224 Dupilumab: 405/447	Screening, baseline, Week 1, 2, 4, 6, 8, 12, EOT (Week 16), follow-up period (Week 20, 24), EOS (Week 28), unscheduled visit, early termination
NCT02260986 R668-AD-1224 [15]	III	Adults (18, 75)	Moderate (IGA = 3) Severe (IGA = 4)	+ TCS	Day 1 loading dose: 2 × maintenance dose • Dupilumab 300 mg SC qw + TCS • Dupilumab 300 mg SC q2w + TCS • Placebo SC qw + TCS	*N* = 740 Dupilumab: 400/425 Placebo: 282/315	Screening, baseline, Week 1, 2, 4, 6, 8, 12, 16, 20, 24, 28, 32, 36, 40, 44, 48, EOT (Week 52), follow-up period (Week 56, 60), EOS (Week 64), unscheduled visit, early termination

^a^Dupilumab dosages are doubled for loading dose administered on Day 1. For consistency, the placebo amount administered on Day 1 was also doubled to match the dupilumab loading doses

^b^The study completion frequencies include only patients who have completed Week 16 of treatment (excludes patients in ongoing studies)

^c^Studies NCT02277769 and NCT02277743 are replicate Phase III clinical trials in adults with moderate-to-severe AD (– TCS)

^d^Patients (< 30 kg) randomly assigned (1:1 ratio) to q2w SC PBO injections matching the 100 mg dupilumab or q4w SC PBO injections matching the 300 mg dupilumab. Patients (≥ 30 kg) randomly assigned (1:1 ratio) to q2w SC PBO injections matching the 200 mg dupilumab or q4w SC PBO injections matching the 300 mg dupilumab. The placebo amount is doubled to match the loading dose on Day 1

^e^IGA and EASI assessment score timepoints are reported from the Study Protocol (not the Clinical Study Report)

AD, atopic dermatitis; EASI, Eczema Area and Severity Index; EOS, end of study; EOT, end of treatment; IGA, Investigator’s Global Assessment; N, number of patients; PBO, placebo; qw, once weekly; q2w, every 2 weeks; q4w, every 4 weeks; SC, subcutaneous; TCS, topical corticosteroids

### Bioanalytical Assay

Serum samples for quantitation of functional dupilumab were analyzed using a validated enzyme-linked immunosorbent assay (ELISA). Dupilumab was used as the assay standard and human IL-4 receptor alpha (IL-4Rα) served as the capture reagent. Concentrations of functional dupilumab with either one or two available binding sites were measured. The lower limit of quantitation (LLOQ) of functional dupilumab is 0.078 mg/L in undiluted human serum [23].

### E-R Model Development

A sequential modeling approach was applied using previously developed population pharmacokinetic analyses performed separately for dupilumab administered in adults (≥ 18 years), adolescents (≥ 12 to < 18 years), and children (≥ 6 to < 12 years) for input to the integrated E-R models [24–26]. Data were continuous, with time-varying concentrations, and data from all the patients were used as part of the dataset for developing the model simulation sets. Models were developed for two efficacy assessments: Eczema Area and Severity Index (EASI) score and Investigator’s Global Assessment (IGA). EASI score was modeled as a continuous variable by transforming the bounded outcome score (range 0–72). IGA was modeled using a latent variable approach, in which the unobserved drug effect was mapped to the probability of response falling in each of the ordered categories (range 0–4; for further details see the Online Resource).

In order to characterize the shape of the placebo effect time course, an empirical model for response was developed using pooled data following placebo treatment across studies and age groups. Based on an understanding of the mechanism of action of dupilumab, an indirect response model with inhibition of k_in_ was evaluated to link efficacy scores and functional dupilumab concentrations, with separate models used per efficacy tool. An I_max_ model was tested to represent maximum inhibitory effects. The E-R relationship was adequately characterized with the I_max_ functional form, thus a sigmoidal model was not considered in the interest of parsimony. Several patient factors, including baseline characteristics and disease severity, were assessed as potential sources of variability on placebo and drug effect parameters and were tested simultaneously to form a full model followed by a stepwise backward elimination procedure. Covariates including baseline demographics (e.g., age, gender, body weight, race), baseline type-2 inflammation markers (e.g., baseline thymus, activation-regulated chemokine [TARC], eosinophil count), TCS co-administration, and prior use of systemic corticosteroids (Online Resource Table S2) were selected based on clinical relevance and mechanistic plausibility. Covariates were evaluated on both placebo and drug effect parameters for both models (Online Resource Table S2). Non-linear mixed-effects modeling methodology was implemented for all steps of this analysis using NONMEM (version 7.3) software (ICON Development Solutions, Ellicott City, MD) [27]. The Laplacian conditional estimation method was used in the IGA analysis and the first-order conditional estimation method with interaction (FOCEI) was used in the EASI analysis. Pre- and post-processing of data and simulations were performed using R software (version 3.6.1, R Foundation for Statistical Computing, Vienna, Austria) [28]. Relative standard error and 95% confidence intervals (CI) were based on the covariance step in NONMEM. Full details of the modeling approach, modeling evaluation, and pharmacokinetic parameter estimates are provided in the supplementary materials (Online Resource, and Table S1, Fig. S1, Fig. S2).

### Model Validation

To evaluate the predictive nature of the models, internal visual predictive checks were performed using the observed dataset used in model development [29]. The observed 5^th^, 50^th^, and 95^th^ percentiles of the EASI efficacy assessment were binned by nominal time and compared to the 90% CI of the simulated efficacy measures at corresponding percentiles. For IGA, the observed responder rate (IGA 0 or 1) was compared to the simulated 90% prediction interval of IGA responder rate. If systematic or major deviations occurred in the model validation process, further model refinement was performed until the predictive performance was adequate.

### Model Applications

#### Application 1: Assessing Individual Covariate Effects

The statistically significant covariates identified from the stepwise backward elimination procedure of the E-R models (see Online Resource for additional details) were further assessed by simulating response for comparator patients differing from a simulated reference subject only in the covariate value being tested, after correcting for differences in placebo effects, holding dupilumab concentrations constant at a therapeutic level (68 mg/L). Further details of the simulations are available in the Online Resource.

The individual effects of covariates on placebo-corrected efficacy, including Week 16 EASI score and the proportion of patients achieving IGA scores of 0 or 1 (IGA 0/1 responders), were evaluated descriptively. For Week 16 placebo-corrected EASI scores, a zero value described no difference in EASI score for a patient on dupilumab and the same patient on placebo; whereas a negative value described a greater reduction of EASI score on dupilumab compared to the same patient on placebo. For Week 16 IGA, the difference in proportion of patients achieving IGA 0 or 1 for patients on dupilumab to patients on placebo was evaluated.

#### Application 2: Comparing E-R across Adults, Adolescents, and Children

In total, 500 clinical trial simulations were performed per scenario to directly compare the E-R relationship across age groups, where factors including baseline disease severity and concomitant TCS therapy were kept constant, and combinations of various patient demographics were incorporated (see Online Resource). This included clinical trial scenarios that were not studied (i.e., children with moderate AD and adolescents receiving dupilumab with TCS co-administration). Placebo-corrected responses were chosen to correct for any differences in placebo response across age groups. Efficacy measures (EASI percent change from baseline, EASI-75, EASI-90, and IGA 0/1) over time were simulated after administration of approved weight-tiered dose regimens. To account for differences in steady-state concentrations across age groups, efficacy was also compared while keeping concentrations constant at each point of the E-R relationship.

## Results

The analysis included a total of 2,968 patients with AD (2,366 adults [> 18 years], 243 adolescents [≥ 12 to < 18 years] and 359 children [≥ 6 to < 12 years]) with 29,413 EASI and 29,420 IGA observations across age groups. Overall, 58.1% of patients were male, 67.2% were White, and 68.2% were given dupilumab without TCS co-administration. The median baseline EASI score was 30.8 (range 10.7–72), and 45.8% and 54.2% patients had moderate (IGA score of 3) and severe AD (IGA score of 4), respectively (Online Resource Table S3).

### Integrated E-R Model Development and Validation

**Fig. 1:**
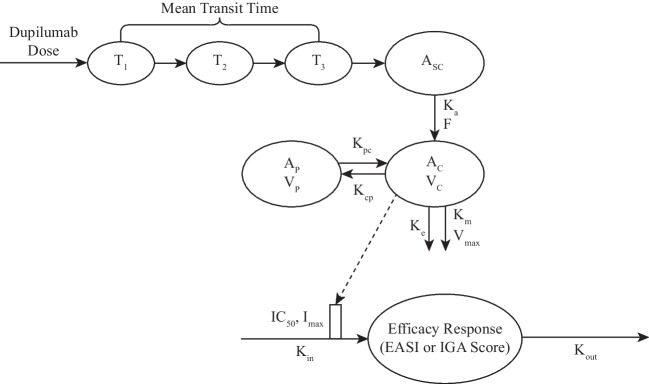
E-R model diagram of dupilumab. A_C_, amount in central compartment; A_SC_, amount in subcutaneous depot compartment; A_P_, amount in peripheral compartment; IC_50_, concentration at which 50% of the maximum effect is achieved; EASI, Eczema Area and Severity Index; E-R, exposure–response; F, bioavailability; IGA, Investigator’s Global Assessment; I_max_, maximum drug effect; k_a_, absorption rate constant; k_in_, rate constant for indirect response production; K_m_, the concentration at which the rate of elimination is half of the maximum value; k_out_, rate constant for indirect response elimination; K_e_, elimination rate constant; K_pc_, K_cp_, intercompartmental rate constants; T, transit compartment; V_max_, the maximum rate of elimination via the non-linear pathway; V_C_, volume of distribution in central compartment; V_P_, volume of distribution in peripheral compartment.

The relationship of the efficacy scores with dupilumab concentration and time was well described by the indirect response model illustrated in Fig. 1. In general, the precision of estimated parameters for both the EASI and IGA models (Tables II and III) was high, with relative standard errors < 4% for structural parameters and < 30% for covariate effects. Based on the estimated time delay of drug response, not accounting for accumulation, the full effect of dupilumab would be reached after approximately 2 months when assessed by EASI and 3 months when assessed by IGA (~ 4–5 half-lives of drug effect onset, see Tables II and III). Drug concentrations achieving half the maximum effect (IC_50_) were lower for the EASI (20.3 mg/L) than IGA (27.1 mg/L) analyses, however the 95% CIs overlapped. Each model had a placebo component indicating some improvement in response measures with time for patients receiving placebo injections.

**Table II:** Parameter estimates of the dupilumab E-R final EASI model

Parameter	EASI model estimate (% RSE)	EASI model (95% CI)
Fixed effects		
Baseline EASI score units	30.4 (0.4)	(29.8, 31.0)
P_max_	−1.24 (2.6)	(−1.30, −1.17)
ET_50_ (days)	29.2	(27.5, 31.0)
Drug effect (I_max_)	0.266 (2.7)	(0.252, 0.280)
IC_50_ (mg/L)	20.3	(16.1, 25.5)
Half-life for drug effect onset (day)	13.7	(12.9, 14.7)
TCS on baseline	−0.0218 (27.9)	(−0.0338, −0.00987)
Prior immunotherapy on baseline	0.0233 (27.5)	(0.0107, 0.0358)
Age on baseline	−0.0372 (13.4)	(−0.0470, −0.0274)
Log baseline eosinophil count on baseline	0.0173 (15.6)	(0.0120, 0.0225)
Log baseline TARC on baseline	0.0508 (4.4)	(0.0464, 0.0552)
TCS on P_max_	0.232 (16.9)	(0.156, 0.309)
Prior immunotherapy on P_max_	−0.166 (18.2)	(−0.225, −0.107)
Baseline body weight on drug effect	−0.185 (23.3)	(−0.270, −0.101)
Asian race on drug effect	−0.374 (8.3)	(−0.434, −0.313)
Log baseline EOS on drug effect	−0.0570 (29.3)	(−0.0897, −0.0242)
Residual variability – additive	0.422 (0.5)	(0.418, 0.426)
IIV		
EASI baseline – exponential (ω^2^)	0.0177 (3.4)	(0.0165, 0.0189)
EASI P_max_ – additive (ω^2^)	0.788 (3.7)	(0.730, 0.846)
OFV	–9277.145	
CN	20.4	

CI, confidence interval; CN, condition number; EASI, Eczema Area and Severity Index; EOS, eosinophil count; E-R, exposure-response; ET_50_, time at which 50% of maximum placebo effect is achieved; IC_50_, concentration at which 50% of the maximum effect is achieved; IIV, interindividual variability; I_max_, maximum drug effect; OFV, objective function value; P_max_, maximum placebo effect; RSE, relative standard error (%); TARC, thymus and activation-regulated chemokine; TCS, topical corticosteroids

Note: ETA shrinkage 11.8% (EASI baseline); 14.3% (EASI P_max_)

**Table III:** Parameter estimates of the dupilumab E-R final IGA score model

Parameter	IGA model estimate (% RSE)	IGA model (95% CI)
Fixed effects		
Baseline for IGA ≤ 3	− 3.35 (3.6)	(− 3.59, − 3.12)
Baseline adjustment for IGA ≤ 2	2.64	(2.59, 2.69)
Baseline adjustment for IGA ≤ 1	1.60	(1.56, 1.63)
Baseline adjustment for IGA ≤ 0	1.75	(1.70, 1.79)
P_max_	4.83 (2.5)	(4.60, 5.06)
ET_50_ (days)	6.37 (3.0)	(5.72, 7.09)
Drug effect (DSLP)	− 2.20 (3.1)	(− 2.33, − 2.07)
IC_50_ (mg/L)	27.1	(21.3, 34.4)
Half-life for drug effect onset (day)	19.7	(18.2, 21.4)
Moderate IGA baseline additive shift	4.39 (3.0)	(4.14, 4.65)
Age on P_max_	0.0550 (21.3)	(0.0320, 0.0780)
TCS on P_max_	0.229 (8.1)	(0.193, 0.266)
Prior immunotherapy on P_max_	− 0.102 (13.1)	(− 0.128, − 0.0756)
Moderate disease severity on P_max_	− 0.727 (1.7)	(− 0.751, − 0.703)
Baseline body weight on drug effect	− 0.174 (26.1)	(− 0.263, − 0.0852)
Asian race on drug effect	− 0.222 (14.2)	(− 0.284, − 0.160)
Log baseline EOS on drug effect	− 0.0776 (19.3)	(− 0.107, − 0.0482)
Log baseline TARC on drug effect	− 0.0938 (13.4)	(− 0.118, − 0.0692)
IIV		
IGA baseline−additive (ω^2^)	1.49 (3.4)	(1.39, 1.59)
OFV	51,593.055	
CN	200	

CI, confidence interval; CN, condition number; DSLP, drug effect slope; EOS, eosinophil count; ET_50_, time at which 50% of maximum placebo effect is achieved; IC_50_, concentration at which 50% of the maximum effect is achieved; IGA, Investigator’s Global Assessment; IIV, interindividual variability; Imax, maximum drug effect; OFV, objective function value; Pmax, maximum placebo effect; RSE, relative standard error (%); TARC, thymus and activation-regulated chemokine; TCS, topical corticosteroids

Note: ETA shrinkage 8.62% (IGA baseline)

**Fig. 2:**
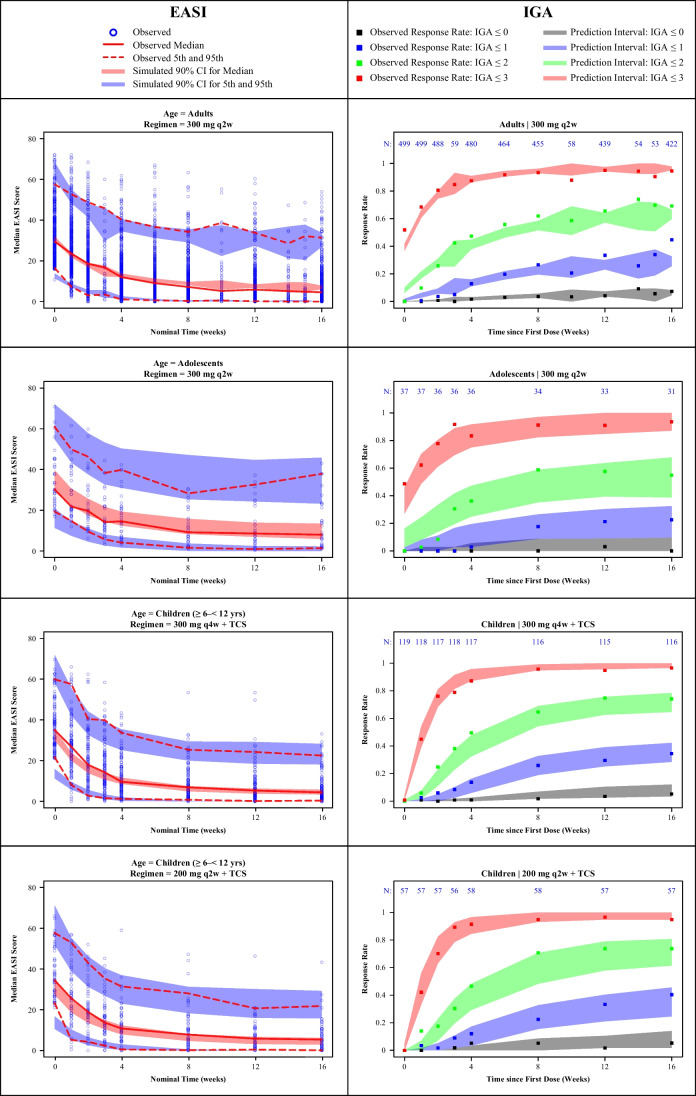
Visual predictive checks for dupilumab approved doses for E-R models of EASI and IGA stratified by age group and treatment regimen. EASI, Eczema Area and Severity Index; E-R, exposure–response; CI, confidence interval; IGA, Investigator’s Global Assessment; qw, once weekly; q2w, every 2 weeks; q4w, every 4 weeks; TCS, topical corticosteroids.

Visual predictive checks stratified by age group (adults, adolescents, and children) and treatment regimen confirmed that both E-R models described the data successfully and were adequate predictive models, as observed responses were largely contained within the 90% CI (EASI) or 90% prediction interval (IGA) (Fig. 2).

### Model Applications

#### Application 1: Assessing Individual Covariate Effects

**Fig. 3:**
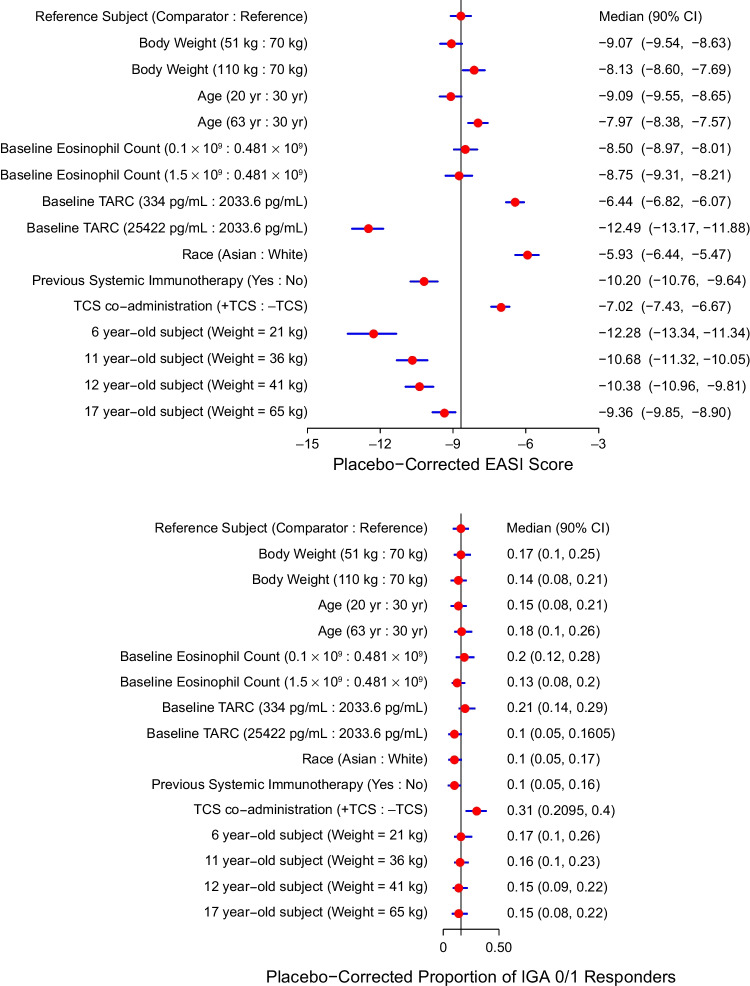
Covariate effects on placebo-corrected EASI scores and placebo-corrected proportions of IGA 0/1 responders. EASI, Eczema Area and Severity Index; IGA 0/1, proportions of patients achieving an Investigator’s Global Assessment score of 0 or 1; q2w, every 2 weeks; TARC, thymus and activation-regulated chemokine; TCS, topical corticosteroids. Note: The vertical line represents the median placebo-corrected response for the reference patient. Red circles represent the median placebo-corrected score under the specified condition (test or reference), and the blue line segments represent the corresponding 90% confidence interval. Test conditions for continuous covariates represent the 5^th^ and 95^th^ percentiles of the covariate in the analysis dataset. Trough dupilumab concentration was fixed to 68 mg/L at Week 16 for all patients. The reference patient for both analyses was defined as White race, baseline body weight 70 kg, age 30 years, no prior systemic immunotherapy, without TCS co-administration, median observed baseline blood eosinophil count (0.481 × 10^9^/L), and median observed TARC serum levels (2033.6 pg/mL).

Simulations of placebo-corrected EASI and IGA responses at Week 16 in reference and comparator patients showed the individual effects of covariates on drug response in the final models (Fig. 3). Younger age, lower weight, higher baseline TARC serum levels, and prior systemic immunotherapy were associated with a greater improvement in placebo-corrected EASI scores compared with the reference patient. However, Asian race was associated with slightly lower improvement in placebo-corrected EASI score compared with the Caucasian reference patient. Paradoxically, TCS co-administration was associated with a slightly lower placebo-corrected EASI response yet a slightly higher IGA response (Fig. 3). In the EASI model, weight, Asian race, and eosinophil count significantly contributed to drug effect, while TCS and prior immunotherapy contributed to placebo effect (Table II). When evaluating response by IGA, covariates that contributed to drug effect were weight, Asian race, eosinophil count, and TARC *versus* the placebo covariates of age, moderate disease, TCS, and prior immunotherapy (Table III).

#### Application 2: Comparing E-R across Adults, Adolescents, and Children

**Fig. 4:**
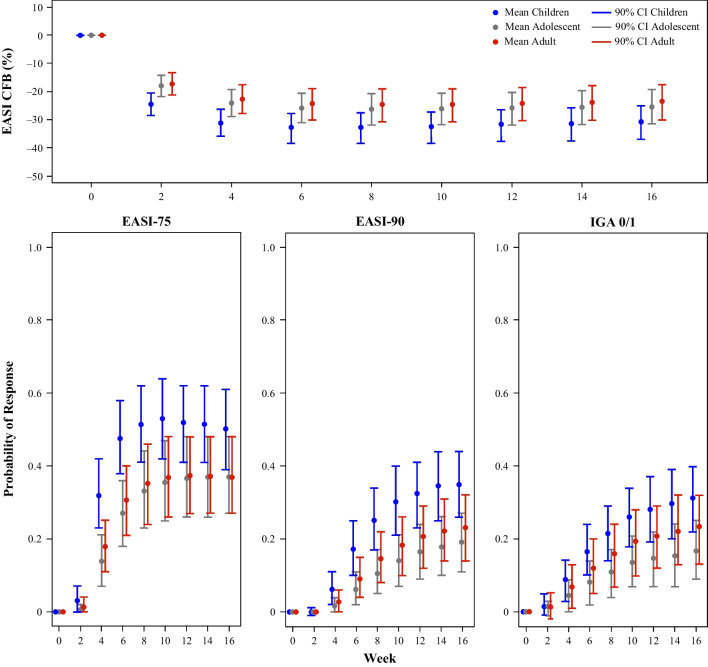
Model-predicted longitudinal efficacy response profiles for dupilumab with TCS in patients with severe disease by age group, corrected for placebo response. CI, confidence interval; CFB, change from baseline; EASI-75/90, ≥ 75%/90% improvement from baseline in Eczema Area and Severity Index scores; IGA, Investigator’s Global Assessment; TCS, topical corticosteroids. Note: Mean (90% CI) represents summary statistics for 500 simulations in each unique combination of categories (age group, disease severity, and TCS co-administration). Dupilumab dosing regimen in adult patients with AD: 600 mg loading dose followed by 300 mg every 2 weeks. Dupilumab dosing regimen in pediatric patients (adolescents and children) with AD: 15 kg to < 30 kg: 600 mg loading dose followed by 300 mg every 4 weeks; 30 kg to < 60 kg: 400 mg loading dose followed by 200 mg every 2 weeks; ≥ 60 kg: 600 mg loading dose followed by 300 mg every 2 weeks (same as adult dose regimen).

Clinical trial simulations that assessed the combined impact of covariates on the efficacy response measures over time after co-administration of approved dose regimens of dupilumab and TCS therapy in severe AD patients are shown in Fig. 4. Across age groups, mean efficacy responses were generally greater for children than for adults and adolescents.

**Fig. 5:**
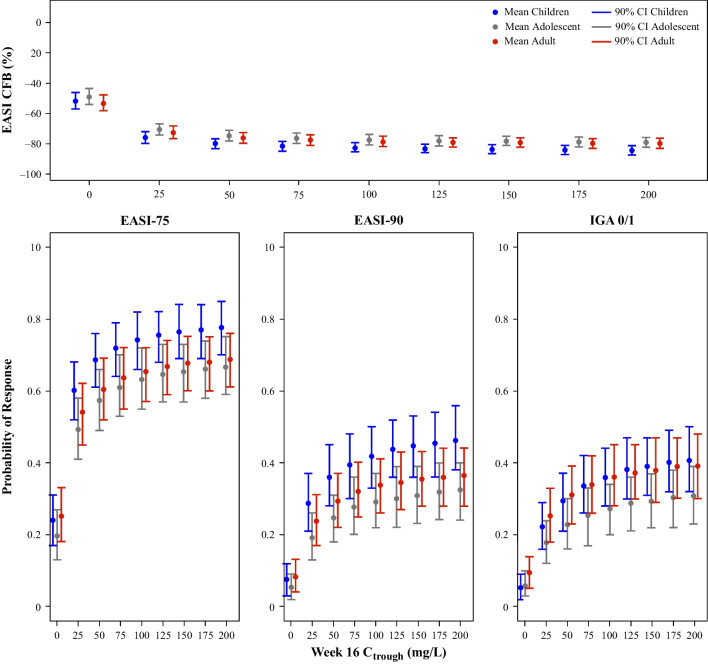
Model-predicted E-R profiles at Week 16 for dupilumab with TCS in patients with severe disease by age group. CI, confidence interval; CFB, change from baseline; C_trough_, dupilumab concentration at the end of a dosing interval; EASI-75/90, ≥ 75%/90% improvement from baseline in Eczema Area and Severity Index scores; E-R, exposure–response; IGA, Investigator’s Global Assessment; TCS, topical corticosteroids. Note: Mean (90% CI) represents summary statistics for 500 simulations in each unique combination of categories (age group, disease severity, and TCS co-administration).

While children had the highest responses, they also had the higher steady-state exposures among the age groups. To assess differential response to treatment at identical exposures, we simulated scenarios where the efficacy could be visualized while maintaining the dupilumab concentration as a constant (Fig. 5). Model-predicted response at Week 16 was summarized across the full E-R relationship and showed that at equivalent dupilumab exposure, children 6–11 years of age had a higher response compared to adults and adolescents (Fig. 5). The difference in response between children and adults/adolescents was associated with increasing baseline disease severity (moderate *versus* severe), but was not related to concomitant TCS administration (Online Resource Figs. S3–S5). Differences between children and adults/adolescents were less pronounced in IGA E-R compared to EASI E-R in patients with severe AD [Figs. 4, 5, S1].

## Discussion

To our knowledge, this is the first E-R analysis to evaluate both categorical (IGA) and continuous (EASI) disease measures using an integrated database across Phase II and III studies in adults, adolescents, and children with moderate-to-severe AD. Multiple clinical trial simulations showed that EASI and IGA responses by drug exposure were generally greater in children, particularly those with severe baseline disease, compared to adults and adolescents.

In this analysis, the response to dupilumab in children was better than in adults or adolescents at similar exposures. This difference may be associated with changes in the underlying disease pathology through life. In addition to differences associated with race [30] and disease severity, immune pathway activation, cytokine and immune cell expression, and clinical presentation of AD have been shown to vary across age groups [31–35]. In a flow cytometry study evaluating the expression of cytokines in skin-homing CD4 and CD8 T cells, Czarnowicki *et al*. demonstrated that children with AD had decreased Th1 cytokine (INF-γ) expression compared to adults with AD [35]. Expression of the Th2 cytokine IL-13 was, however, similar between the age groups. Those data, which suggest that reduced counter-regulation by Th1 T cells may contribute to excess Th2 activation in children compared to adults, are consistent with the slightly higher drug response in children *versus* adults observed in the current analysis. Proteomic analyses of blood samples have also shown systemic Th2 skewing and different expression patterns in pediatric and adult AD, although gene expression analyses in blood and skin have identified largely different marker sets [30, 36].

Older age, higher body weight, lower baseline TARC (a correlate of baseline disease severity), and Asian race were associated with lesser improvements in EASI in response to dupilumab; however, these trends were not observed in IGA response. Covariates that were significant on drug effect, as measured by EASI, were weight, Asian race, and eosinophil count, *versus* effects of TCS and prior immunotherapy on placebo. For response by IGA, covariates that contributed to drug effect were weight, Asian race, eosinophil count, and TARC *versus* the placebo effect covariates of age, moderate disease, TCS, and prior immunotherapy. Differences in identified covariates between EASI and IGA can be attributed to the fact that the scales are different: EASI measures both extent and severity of disease, while IGA measures disease lesional severity. Nevertheless, combinations of covariates affecting response in the same direction could potentially be considered when evaluating patients who are relatively less responsive to treatment. Surprisingly, TCS co-treatment appeared to be associated with lower EASI but higher IGA response, a result that would be of interest to investigate further in future studies.

In general, continuous efficacy assessments may be more sensitive to drug treatment than categorical ones, hence the slightly higher (albeit similar) IC_50_ estimate in the IGA model. The estimates of IC_50_ are supported by dupilumab concentrations associated with clinical efficacy. Typically, approximately four multiples of the IC_50_ result in concentrations associated with near maximal drug effect in the hill function when the sigmoidicity constant ($\gamma$) is one. Based on this, the IC_50_ estimates of both scales are consistent with therapeutic concentrations of dupilumab in adult and pediatric patients with AD (70–100 mg/L).

Previous E-R analysis of dupilumab in children and adolescents based on non-linear logistic regression and scatterplots analysis of categorical and continuous efficacy scores, respectively, showed a relationship of increased efficacy with increasing dupilumab concentrations in these age groups [22]. The current analysis has advantages over these previous E-R assessments in pediatric patients with AD in that it was integrated across adults and pediatric age groups and employed a non-linear mixed-effects methodology, providing a non-confounded, direct comparison across age groups, accounting for covariates of response. It also used data collected throughout the study, whereas previous assessments were limited to Week 16 data. The integrated analysis allowed evaluation across the differing study designs (e.g., monotherapy vs TCS co-administration), placebo response rates, and baseline disease severity. It also enabled simulation of clinical scenarios which were not studied. This feature was particularly informative as the label indication for dupilumab extends to patients outside the inclusion criteria for the included Phase II and III clinical trials (e.g., in adolescents together with TCS, and children with moderate AD).

We acknowledge a number of limitations to this analysis. Due to the collinearity of age and body weight in younger children, the differences in E-R between children and adults/adolescents may not be solely attributed to age. Also, assumptions of covariate correlations with disease severity were necessary for clinical trial simulations as some of the scenarios were not studied (e.g., moderate baseline disease severity in children). When available, data from pediatric patients with moderate AD should be used to validate the assumptions made. Furthermore, data from pediatric patients < 6 years of age were not available at the time of the analysis, therefore future applications of this model should include evaluations in this age group. A final limitation of the current study is that it only evaluated relationships between efficacy and systemic drug exposure, and therefore may not be fully representative of differences at a tissue level.

In the US, dupilumab is approved for adult and pediatric patients with AD and asthma, and in adults with chronic rhinosinusitis with nasal polyps, eosinophilic esophagitis, and prurigo nodularis [6]. It is currently being investigated in pediatric patients with type 2 inflammatory diseases, including chronic spontaneous urticaria and chronic rhinosinusitis with nasal polyps, and in young children with eosinophilic esophagitis. Because this analysis demonstrated similar (or better) E-R in pediatric patients compared with adults in AD, pharmacokinetic bridging from adults to pediatric patients may be justified. This is consistent with the U.S Food and Drug Administration and International Council for Harmonisation of Technical Requirements for Pharmaceuticals for Human Use guidance that detail the criteria for bridging by pharmacokinetics from a reference population to pediatric patients, in combination with adequate collection of safety data in the target population [37, 38]. Therefore, as future type 2 inflammatory indications of dupilumab are pursued, data from adult studies may be leveraged to extrapolate expected responses to dupilumab in younger children where large prospective, randomized, controlled pediatric trials assessing efficacy may be difficult to generate.

## Conclusion

In summary, we developed an integrated E-R model of dupilumab in children, adolescents, and adults with moderate-to-severe AD, using both categorical and continuous endpoints. This modeling approach may be generalizable to some of the other type 2 inflammatory diseases with similar pathophysiology.

### Supplementary Information

Below is the link to the electronic supplementary material.

Supplementary file1 (DOCX 5.35 MB)

## Data Availability

Qualified researchers may request access to study documents (including the clinical study report, study protocol with any amendments, blank case report form, and statistical analysis plan) that support the methods and findings reported in this manuscript. Individual anonymized participant data will be considered for sharing once the indication has been approved by a regulatory body, if there is legal authority to share the data and there is not a reasonable likelihood of participant re-identification. Submit requests to https://vivli.org/.

## References

[1] Laughter MR, Maymone MBC, Mashayekhi S, Arents BWM, Karimkhani C, Langan SM (2021). The global burden of atopic dermatitis: lessons from the Global Burden of Disease Study 1990–2017. Br J Dermatol.

[2] Kowalska-Olędzka E, Czarnecka M, Baran A (2019). Comparison of treatment standards in atopic dermatitis management across selected geographies prior to emerging targeted therapies onset. J Drug Assess.

[3] Eichenfield LF, Tom WL, Berger TG, Krol A, Paller AS, Schwarzenberger K, *et al*. Guidelines of care for the management of atopic dermatitis: section 2. Management and treatment of atopic dermatitis with topical therapies [published online ahead of print, 2023 Jan 12]. J Am Acad Dermatol. 2014;71(1):116–32. 10.1016/j.jaad.2014.03.023 PMC4326095 24813302

[4] Sidbury R, Davis DM, Cohen DE, Cordoro KM, Berger TG, Bergman JN, *et al*. Guidelines of care for the management of atopic dermatitis: section 3. Management and treatment with phototherapy and systemic agents. J Am Acad Dermatol. 2014;71(2):327–49. 10.1016/j.jaad.2014.03.030 PMC4410179 24813298

[5] Sidbury R, Alikhan A, Bercovitch L, Cohen DE, Darr JM, Drucker AM, *et al*. Guidelines of care for the management of atopic dermatitis in adults with topical therapies. J Am Acad Dermatol. 2023. 10.1016/j.jaad.2022.12.029. 10.1016/j.jaad.2022.12.029 36641009

[6] Boguniewicz M, Alexis AF, Beck LA, Block J, Eichenfield LF, Fonacier L (2017). Expert perspectives on management of moderate-to-severe atopic dermatitis: a multidisciplinary consensus addressing current and emerging therapies. J Allergy Clin Immunol Pract.

[7] DUPIXENT (dupilumab). Highlights of Prescribing Information. FDA, Updated October 2022. https://www.regeneron.com/downloads/dupixent_fpi.pdf. Accessed 18 Mar 2023.

[8] Macdonald LE, Karow M, Stevens S, Auerbach W, Poueymirou WT, Yasenchak J (2014). Precise and in situ genetic humanization of 6 Mb of mouse immunoglobulin gene. Proc Natl Acad Sci U S A.

[9] Murphy AJ, Macdonald LE, Stevens S, Karow M, Dore AT, Pobursky K (2014). Mice with megabase humanization of their immunoglobulin genes generate antibodies as efficiently as normal mice. Proc Natl Acad Sci U S A.

[10] Le Floc'h A, Allinne J, Nagashima K, Scott G, Birchard D, Asrat S (2020). Dual blockade of IL-4 and IL-13 with dupilumab, an IL-4Rα antibody, is required to broadly inhibit type 2 inflammation. Allergy.

[11] Paller AS, Siegfried EC, Thaçi D, Wollenberg A, Cork MJ, Arkwright PD (2020). Efficacy and safety of dupilumab with concomitant topical corticosteroids in children 6 to 11 years old with severe atopic dermatitis: a randomized, double-blinded, placebo-controlled phase 3 trial. J Am Acad Dermatol.

[12] Simpson EL, Paller AS, Siegfried EC, Boguniewicz M, Sher L, Gooderham MJ (2020). Efficacy and safety of dupilumab in adolescents with uncontrolled moderate to severe atopic dermatitis: a phase 3 randomized clinical trial. JAMA Dermatol.

[13] Thaçi D, Simpson EL, Beck LA, Bieber T, Blauvelt A, Papp K (2016). Efficacy and safety of dupilumab in adults with moderate-to-severe atopic dermatitis inadequately controlled by topical treatments: a randomised, placebo-controlled, dose-ranging phase 2b trial. Lancet.

[14] Simpson EL, Bieber T, Guttman-Yassky E, Beck LA, Blauvelt A, Cork MJ (2016). Two phase 3 trials of dupilumab versus placebo in atopic dermatitis. N Engl J Med.

[15] Blauvelt A, de Bruin-Weller M, Gooderham M, Cather JC, Weisman J, Pariser D (2017). Long-term management of moderate-to-severe atopic dermatitis with dupilumab and concomitant topical corticosteroids (LIBERTY AD CHRONOS): a 1-year, randomised, double-blinded, placebo-controlled, phase 3 trial. Lancet.

[16] Castro M, Corren J, Pavord ID, Maspero J, Wenzel S, Rabe KF (2018). Dupilumab efficacy and safety in moderate-to-severe uncontrolled asthma. N Engl J Med.

[17] Rabe KF, Nair P, Brusselle G, Maspero JF, Castro M, Sher L (2018). Efficacy and safety of dupilumab in glucocorticoid-dependent severe asthma. N Engl J Med.

[18] Bacharier LB, Maspero JF, Katelaris CH, Fiocchi AG, Gagnon R, de Mir I (2021). Dupilumab in children with uncontrolled moderate-to-severe asthma. N Engl J Med.

[19] Bachert C, Han JK, Desrosiers M, Hellings PW, Amin N, Lee SE (2019). Efficacy and safety of dupilumab in patients with severe chronic rhinosinusitis with nasal polyps (LIBERTY NP SINUS-24 and LIBERTY NP SINUS-52): results from two multicentre, randomised, double-blind, placebo-controlled, parallel-group phase 3 trials. Lancet.

[20] Dellon ES, Rothenberg ME, Collins MH, Hirano I, Chehade M, Bredenoord A (2022). Dupilumab in adults and adolescents with eosinophilic esophagitis. N Engl J Med.

[21] Yosipovitch G, Mollanazar N, Ständer S, Kwatra SG, Kim BS, Laws E, *et al*. Dupilumab in patients with prurigo nodularis: two randomized, double-blind, placebo-controlled phase 3 trials [published online ahead of print, 2023 May 4]. Nat Med. 2023. 10.1038/s41591-023-02320-9. 10.1038/s41591-023-02320-9 PMC10202800 37142763

[22] Kamal MA, Kovalenko P, Kosloski MP, Srinivasan K, Zhang Y, Rajadhyaksha M (2021). The posology of dupilumab in pediatric patients with atopic dermatitis. Clin Pharmacol Ther.

[23] Davis JD, Bansal A, Hassman D, Akinlade B, Li M, Li Z (2018). Evaluation of potential disease-mediated drug-drug interaction in patients with moderate-to-severe atopic dermatitis receiving dupilumab. Clin Pharmacol Ther.

[24] Kovalenko P, DiCioccio AT, Davis JD, Li M, Ardeleanu M, Graham N (2016). Exploratory population PK analysis of dupilumab, a fully human monoclonal antibody against IL-4Rα, in atopic dermatitis patients and normal volunteers. CPT Pharmacometrics Syst Pharmacol.

[25] Center for Drug Evaluation and Research. Approval package for dupixent (dupilumab). Available from: https://www.accessdata.fda.gov/drugsatfda_docs/nda/2022/761055Orig1s012.pdf. Accessed 11 May 2023.

[26] BLA multi-disciplinary review and evaluation of dupixent (dupilumab). Available from: https://www.fda.gov/media/139657/download. Accessed 10 May 2023.

[27] Beal S, Sheiner LB, Boeckmann A, Bauer RJ. NONMEM User's Guides. (1989–2016). Ellicott City: Icon Development Solutions; 2016.

[28] R Core Team. R: A language and environment for statistical computing. R Foundation for Statistical Computing. Vienna, Austria. https://www.r-project.org/. Accessed 13 Oct 2023.

[29] Bergstrand M, Hooker AC, Wallin JE, Karlsson MO (2011). Prediction-corrected visual predictive checks for diagnosing nonlinear mixed-effects model. AAPS J.

[30] Brunner PM, Guttman-Yassky E (2019). Racial differences in atopic dermatitis. Ann Allergy Asthma Immunol.

[31] Bieber T (2010). Atopic dermatitis. Ann Dermatol.

[32] Ständer S (2021). Atopic dermatitis. N Engl J Med.

[33] Yew YW, Thyssen JP, Silverberg JI (2019). A systematic review and meta-analysis of the regional and age-related differences in atopic dermatitis clinical characteristics. J Am Acad Dermatol.

[34] Renert-Yuval Y, Del Duca E, Pavel AB, Fang M, Lefferdink R, Wu J (2021). The molecular features of normal and atopic dermatitis skin in infants, children, adolescents, and adults. J Allergy Clin Immunol.

[35] Czarnowicki T, Esaki H, Gonzalez J, Malajian D, Shemer A, Noda S (2015). Early pediatric atopic dermatitis shows only a cutaneous lymphocyte antigen (CLA)(+) TH2/TH1 cell imbalance, whereas adults acquire CLA(+) TH22/TC22 cell subsets. J Allergy Clin Immunol.

[36] Brunner PM, Israel A, Leonard A, Pavel AB, Kim HJ, Zhang N (2019). Distinct transcriptomic profiles of early-onset atopic dermatitis in blood and skin of pediatric patients. Ann Allergy Asthma Immunol.

[37] US Food and Drug Industry. Clinical Pharmacology Considerations for Pediatric Studies of Drugs, Including Biological Products. 2022. https://www.fda.gov/regulatory-information/search-fda-guidance-documents/general-clinical-pharmacology-considerations-pediatric-studies-drugs-including-biological-products. Accessed 13 Oct 2023.

[38] International Council for Harmonisation of Technical Requirements for Pharmaceuticals for Human Use. ICH Harmonised Guideline: Pediatric Extrapolation E11A. 2022. https://www.fda.gov/media/161190/download. Accessed 13 Oct 2023.

