# Supplementary material for: Integrated Exposure–Response of Dupilumab in Children, Adolescents, and Adults With Atopic Dermatitis Using Categorical and Continuous Efficacy Assessments: A Population Analysis
Source: Pharm Res. 2023 Dec 11;40(11):2653–66. doi: 10.1007/s11095-023-03616-8 (PMC10733507; doi:10.1007/s11095-023-03616-8)
Supplement: Supplementary file 1 — Supplementary file1 (DOCX 5.35 MB) [file 11095_2023_3616_MOESM1_ESM.docx]

**Supplementary Materials**

**Title: Integrated Exposure**–**Response of Dupilumab in Children, Adolescents, and Adults With Atopic Dermatitis Using Categorical and Continuous Efficacy Assessments: A Population Analysis**

Emily Briggs **·** Mohamed A. Kamal **·** Matthew P. Kosloski **·** Ian Linsmeier **·** Natalie Jusko **·**
Nancy Dolphin **·** Jason Chittenden **·** Eric L. Simpson **·** Amy S. Paller **·** Elaine C. Siegfried **·** Brad Shumel **·** Noah A. Levit **·** Ashish Bansal **·** John D. Davis **·** Sunny Chapel **·** David E. Smith **·** Nidal Huniti

**Supplementary Methods**

**Population Pharmacokinetic Model**

The population pharmacokinetic models consisted of two-compartment disposition with parallel linear and non-linear (Michaelis–Menten) elimination and three transit compartments describing absorption. Post-dose samples below the lower limit of quantitation were excluded. Individual predicted PK parameters were incorporated when fitting the exposure–response (E-R) model, along with actual dosing histories of each patient and efficacy observations.

**Eczema Area and Severity Index (EASI)**

The EASI is a validated measure used in clinical practice and clinical trials to assess the severity and extent of atopic dermatitis (AD). The EASI score calculation is based on the Physician’s Assessment of Individual Signs (erythema [E], induration/papulation [I], excoriation [X], and lichenification [L]), where each sign is scored as 0 = absent, 1 = mild, 2 = moderate, or 3 = severe, and also upon the Area Score (based on the % body surface area [BSA] affected), where 0 = 0% BSA, 1 = 1 to 9% BSA, 2 = 10 to 29% BSA, 3 = 30 to 49% BSA, 4 = 50 to 69% BSA, 5 = 70 to 89% BSA, and 6 = 90 to 100% BSA (1).

For each major section of the body (head, upper extremities, trunk, and lower extremities), EASI score = (E+I+X+L) x Area Score. The total EASI score is the weighted total of the section EASI using the weights 10% = head, 20% = upper extremities, 30% = trunk, and 40% = lower extremities in patients ≥ 8 years of age, and 20% = head, 20% = upper extremities, 30% = trunk, and 30% = lower extremities in patients < 8 years of age. The minimum possible EASI score is 0; the maximum possible EASI score is 72, with a higher score indicating increased extent and severity of AD.

**Investigator’s Global Assessment (IGA)**

The IGA score is a 5-point scale (ranging from 0 to 4) to assess AD disease severity, where higher scores indicate greater severity. The IGA uses clinical characteristics, such as erythema, lichenification, and oozing, to assess disease severity (2). Patient-reported outcomes, such as pruritus symptoms, were not evaluated in this analysis.

**Indirect Response Model**

The functional form of the EASI response model was specified as:

$$EASI=f_{baseline}+f_{non-drug}\left( t \right)+f_{drug}\left( E\left( t \right) \right)+\varepsilon$$

where *f_baseline_* is the baseline component, *f_non-drug_* is the non-drug (placebo) time component, *f_drug_* is the drug (exposure) component, *E*(*t*) represents drug exposure as a function of time (*t*), and *ε* is the residual error assumed to be normally distributed with mean 0 and variance equal to $\sigma^{2}$ (i.e., *ε*~ *N*(0, $\sigma^{2}$)). Tukey’s transformation was applied to normalize observed EASI data, and the model was postulated on the transformed scale: (𝐸𝐴𝑆𝐼 + 1)^λ^ where λ = 0.4.

Indirect response models have been well characterized in the literature (3) and used when there is a temporal delay between peak exposure and maximum response. The models are represented through an inhibition of k_in_ (Type I indirect response model):

$$\frac{dR}{dt} = k_{in}\cdot\left( 1- \frac{Imax\cdot C(t)}{{IC}_{50}+C(t)} \right)-k_{out}\cdot R$$

where *R* denotes the response variable with initial condition R_0_ = k_in_/k_out_ = BASE, *k_in_* is the rate constant for indirect response production, *k_out_* is the rate constant for indirect response elimination, *Imax* is the maximum inhibitory drug effect, *IC_50_* is the concentration at which 50% of the maximum effect is achieved, and *C(t)* is the drug concentration at time *t*.

Given the ordered categorical nature of IGA scores, an extension of the indirect response model was applied by incorporating a latent variable component. The latent variable is an unobservable variable that can be mapped to ordered categorical data (4). The following form was used to represent the model, posited on the probit scale:

$$probit Pr\left( IGA\leq m \right)=f_{baseline}\left( m \right)+f_{non-drug}\left( t \right)+f_{drug}\left( E\left( t \right) \right)$$

$$\frac{dR}{dt}=k_{out}\cdot\left[ 1-\frac{C(t)}{{IC}_{50}+C(t)} \right]-k_{out}\cdot(R+1), k_{out}=\frac{ln \left( 2 \right)}{THFD}$$

where the terms *f_baseline_*, *f_non-drug_*, and *f_drug_* represent the baseline, non-drug (or placebo), and drug functions, respectively, *t* represents time, and *E*(*t*) represents drug exposure as a function of time. *R*, *k_out_*, *IC_50_*, and *C(t)* are defined as above, and THFD represents the half-life for drug effect onset. The model was defined using the probit scale, where $probit=\Phi^{-1}$, $\Phi\left( \cdot\right)$ is the cumulative normal distribution function and *m* represents the observed IGA score.

The baseline $f_{baseline}\left( m \right)$ component is defined recursively as:

$$f_{baseline}\left( m \right) =Q_{m}; Q_{m}=\{BASE m=3 Q_{m}=Q_{m+1}-exp\left( \beta_{m+1} \right) 0\leq m\leq2$$

where BASE is the baseline function for an IGA value of 3 and $\beta_{m+1}$, $m\in\left\{ 0,1,2 \right\}$ are parameters that adjust the thresholds for the corresponding observed IGA values.

For both endpoints, interindividual variability was modeled using a log-normal distribution, and the residual error was described using an additive error model. Covariates evaluated as part of the full covariate model are described in Table S2. The backward elimination procedure was associated with a significance level of α=0.001.

The final model equations for each model are summarized below.

EASI model equations:

$$\left( EASI+1 \right)^{\lambda}=BASE + \frac{P_{max}*Time}{ET_{50}+Time} + IDR , \lambda=0.4$$

$$\frac{dR}{dt}=k_{in} \cdot\left[ 1-\frac{E_{max}\cdot C}{{EC}_{50}+C} \right]-k_{out}\cdot R, k_{in}=k_{out}*BASE$$

where IDR = indirect response function; *P_max_* = maximum placebo effect; *ET_50_* is the time at which there is 50% of maximum placebo effect; R = response; k_in_ = rate constant for indirect response production; k_out_ = rate constant for indirect response elimination; C = dupilumab concentration.

IGA model equations:

$$probit Pr\left( IGA\leq m \right)=Q_{m} +\frac{P_{max}*Time}{ET_{50}+Time}+DSLP*R$$

$$Q_{m}=\{BASE m=3 Q_{m}=Q_{m+1}-exp\left( \beta_{m+1} \right) 0\leq m\leq2$$

$$\frac{dR}{dt}=k_{out}\cdot\left[ 1-\frac{C}{{EC}_{50}+C} \right]-k_{out}\cdot(R+1), k_{out}=\frac{ln\left( 2 \right)}{THFD}$$

where BASE is the baseline function for an IGA value of 3 and $\beta_{m+1}$, $m\in\left\{ 0,1,2 \right\}$ are parameters that adjust the thresholds for the corresponding observed IGA values; *P_max_* = maximum placebo effect; *ET_50_* is the time at which there is 50% of maximum placebo effect; k_out_ = rate constant for indirect response elimination; C = dupilumab concentration; THFD = half-life for drug effect onset (day).

### Population E-R Model Evaluation

Models were assessed using the Akaike information criterion, goodness-of-fit plots, precision of parameter estimates, and stability of the model. To avoid ill-conditioning, inspection of the covariance matrix of estimates at every stage of model development was performed to verify that extreme pairwise correlations (*ρ* > 0.95) of the parameters were not encountered. The condition number was also assessed to ensure values less than 1000, above which would indicate a severely ill-conditioned model (5). Inspection of standard diagnostic plots suggested good agreement between observed and population-predicted response for both the pooled population and population stratified by age classification (children, adolescents, and adults; Fig. S1, Fig. S2).

### Model Application 1: Additional Details

A simulated comparator subject was created for each covariate condition differing from the reference subject only in the covariate value being tested. To calculate the proportion of patients with positive IGA response (0 or 1), 100 patients were simulated per group. Differences in placebo-corrected EASI score or IGA response rates between comparator and reference conditions were calculated for each covariate.

### Model Application 2: Additional Details

A total of 500 simulation datasets were created, which replicated the designs, subject populations, dose regimens, sample sizes, and covariate distributions of the pooled analysis dataset. Each dataset consisting of 100 virtual patients per age group (adults, adolescents, and children) and disease severity (baseline IGA of 3 or 4). The simulation datasets were constructed by sampling with replacement from among the dupilumab-treated patients in the analysis dataset along with their complete covariate vectors. Since children with moderate disease severity were not available in the analysis dataset, subjects were sampled from among the children with severe disease and assigned eosinophil count and thymus and activation-regulated chemokine (TARC) from adolescents with moderate disease severity to create a set of virtual patients of age 6–12 years (children) with moderate disease severity, as eosinophil count and TARC were found to be correlated with disease severity. The same virtual patients were administered placebo and dupilumab treatments in the simulations, and each treatment was given alone and with topical corticosteroids.

**Table S1.** Final PK parameter estimates (6).

| Parameter name | Children  ≥ 6 to < 12 years | Adolescents  ≥ 12 to < 18 years | Adults  ≥ 18 years |
| --- | --- | --- | --- |
| PK parameter | | | |
| V_2_ (L) | 2.18 (0.0872) | 2.47 (0.0501) | 2.74 (0.021) |
| K_e_ (1/day) | 0.0446 (0.00152) | 0.0520 (0.00188) | 0.0477 (0.00078) |
| V_m_ (mg/L/day) | 1.64 (fixed) | 1.43 (0.0379) | 1.07 (fixed) |
| K_23_ (1/day) | 0.211 (fixed) | 0.211 (fixed) | 0.211 (fixed) |
| K_32_ (1/day) | 0.310 (fixed) | 0.310 (fixed) | 0.310 (fixed) |
| K_a_ (1/day) | 0.641 (fixed) | 0.306 (fixed) | 0.306 (fixed) |
| MTT (day) | 0.105 (fixed) | 0.105 (fixed) | 0.105 (fixed) |
| K_m_ (mg/L) | 0.01 (fixed) | 0.01 (fixed) | 0.01 (fixed) |
| F (unitless) | 0.642 (fixed) | 0.642 (fixed) | 0.642 (fixed) |
| Covariates | | | |
| V_2_ ~ Weight | 0.849 (0.0345) | 0.755 (0.0517) | 0.817 (0.031) |
| V_2_ ~ Albumin | −0.525 (0.149) | --- | −0.653 (0.072) |
| K_e_ ~ BMI | --- | 0.357 (0.116) | 0.368 (0.053) |
| K_e_ ~ ADA | --- | 0.193 (0.05666) | 0.164 (0.029) |
| K_e_ ~ EASI | 0.169 (0.0471) | 0.356 (0.0523) | 0.143 (0.021) |
| K_e_ ~Race (White) | --- | --- | −0.123 (0.018) |
| Omega matrix | | | |
| $\sigma(lnln \left( V_{2} \right) )$ | 0.291 (0.0204) | 0.140 (0.0145) | 0.206 (0.0068) |
| $\sigma(lnln \left( K_{e} \right) )$ | 0.417 (0.0282) | 0.304 (0.0242) | 0.293 (0.010) |
| Corr ($ln \left( K_{e} \right) ,ln (V_{2}))$ | −0.883 (0.0212) | −0.529 (0.0902) | −0.450 (0.035) |
| Residual SD | | | |
| $\sigma_{prop}$ (CV%) | 13.1 (0.402) | 9.94 (0.602) | 12.5 (0.18) |
| $\sigma_{add}$ (mg/L) | 0.03 (fixed) | 2.36 (0.24) | 6.06 (0.23) |
| Derived parameters | | | |
| CL (L/day) | 0.0972 | 0.128 | 0.131 |
| Q (L/day) | 0.460 | 0.521 | 0.578 |
| V_3_ (L) | 1.48 | 1.68 | 1.86 |

ADA, anti-drug antibody; BMI, body mass index; CL, clearance rate; CV, coefficient of variation; EASI, Eczema Area and Severity Index; F, bioavailability; K_23_, K_32_, inter-compartmental rate constants; K_a_, absorption rate constant; K_e_, elimination rate constant; K_m_, Michaelis–Menten constant; MTT, mean transit time; PK, pharmacokinetic; Q, blood flow; SD, standard deviation; V_2_, central compartment volume; V_3_, peripheral compartment volume; V_m_, maximum target-mediated rate of elimination.

**Table S2.** Covariates evaluated for the EASI and IGA models.

| **Model** | **Parameter** | **Covariates evaluated** |
| --- | --- | --- |
| EASI | Baseline EASI Score | Body weight, age, TCS co-administration, prior exposure of systemic immunotherapies, baseline eosinophil count, baseline TARC |
|  | Non-Drug (Placebo) Effect Parameter | Body weight, age, race (Asian, Black, Other), TCS co-administration, prior exposure of systemic immunotherapies |
|  | Drug Effect Parameter | Body weight, age, race (Asian, Black, Other), prior exposure of systemic immunotherapies, baseline eosinophil count, baseline TARC |
| IGA | Non-Drug (Placebo) Effect Parameter | Body weight, age, race (Asian, Black, Other), TCS co-administration, prior exposure of systemic immunotherapies, disease severity (IGA) |
|  | Drug Effect Parameter | Body weight, age, race (Asian, Black, Other), prior exposure of systemic immunotherapies, baseline eosinophil count, baseline TARC, disease severity (IGA) |

EASI, Eczema Area and Severity Index; IGA, Investigator’s Global Assessment; TARC, thymus and activation-regulated chemokine; TCS, topical corticosteroids.

**Table S3.** Summary of baseline disease status and treatment variables for the patients included in the population E-R analysis, by age group.

| Covariate | Adults | Adolescents | Children | Total |
| --- | --- | --- | --- | --- |
| Patients (n) | 2366 | 243 | 359 | 2968 |
| Age (years), mean (SD) | 37.7 (13.7) | 14.4 (1.7) | 8.5 (1.7) | 32.2 (16.4) |
| Body weight (kg), mean (SD) | 76.1 (18.5) | 65.0 (21.8) | 31.5 (10.4) | 69.8 (23.2) |
| Body mass index (kg/m^2^), mean (SD) | 26.1 (5.7) | 24.3 (6.6) | 17.8 (3.6) | 25.0 (6.2) |
| Body surface area (m^2^), mean (SD) | 1.89 (0.26) | 1.70 (0.31) | 1.06 (0.21) | 1.77 (0.37) |
| Sex |  |  |  |  |
| Male, n (%) | 1,403 (59.3%) | 143 (58.8%) | 179 (49.9%) | 1725 (58.1%) |
| Female, n (%) | 963 (40.7%) | 100 (41.2%) | 180 (50.1%) | 1243 (41.9%) |
| Race |  |  |  |  |
| White, n (%) | 1592 (67.3%) | 154 (63.4%) | 248 (69.1%) | 1994 (67.2%) |
| Black, n (%) | 150 (6.3%) | 29 (11.9%) | 60 (16.7%) | 239 (8.1%) |
| Asian, n (%) | 561 (23.7%) | 36 (14.8%) | 28 (7.8%) | 625 (21.1%) |
| Native Hawaiian or other  Pacific Islander, n (%) | 3 (0.1%) | 3 (1.2%) | 1 (0.3%) | 7 (0.2%) |
| American Indian or  Alaska Native, n (%) | 2 (0.1%) | 1 (0.4%) | 1 (0.3%) | 4 (0.1%) |
| Other, n (%) | 41 (1.7%) | 15 (6.2%) | 17 (4.7%) | 73 (2.5%) |
| Not reported, n (%) | 17 (0.7%) | 5 (2.1%) | 4 (1.1%) | 26 (0.9%) |
| Japanese ethnicity |  |  |  |  |
| No, n (%) | 2099 (88.7%) | 243 (100%) | 359 (100%) | 2701 (91.0%) |
| Yes, n (%) | 267 (11.3%) | 0 (0%) | 0 (0%) | 267 (9.0%) |
| Prior exposure to systemic immunotherapies |  |  |  |  |
| No, n (%) | 1726 (73.0%) | 134 (55.1%) | 171 (47.6%) | 2023 (68.2%) |
| Yes, n (%) | 40 (27.0%) | 109 (44.9%) | 188 (52.4%) | 945 (31.8%) |
| TCS co-administration |  |  |  |  |
| No, n (%) | 1667 (70.5%) | 243 (100%) | 0 (0%) | 1910 (64.4%) |
| Yes, n (%) | 699 (29.5%) | 0 (0%) | 359 (100%) | 1058 (35.6%) |
| Baseline IGA score |  |  |  |  |
| Moderate AD (IGA=3) | 1245 (52.6%) | 112 (46.1%) | 1 (0.3%) | 1358 (45.8%) |
| Severe AD (IGA=4) | 1121 (47.4%) | 131 (53.9%) | 358 (99.7%) | 1610 (54.2%) |
| Baseline EASI score, mean (SD) | 32.6 (13.4) | 35.5 (14.1) | 37.9 (11.7) | 33.5 (13.4) |
| Baseline TARC (pg/mL), mean (SD) | 6625 (13,576) | 5960 (9,414) | 3511 (6,967) | 6203 (12,706) |
| Minimum | 58.4 | 183 | 15.7 | 15.7 |
| Baseline EOS (x10^9^/L), mean (SD) | 0.58 (0.55) | 0.83 (0.65) | 0.83 (0.62) | 0.63 (0.57) |
| Treatment received |  |  |  |  |
| Placebo, n (%) | 801 (33.9) | 83 (34.2) | 120 (33.4) | 1004 (33.8) |
| Dupilumab, n (%) | 1565 (66.1) | 160 (65.8) | 239 (66.6) | 1964 (66.2) |
| Number of observations |  |  |  |  |
| EASI, n (%) | 24,921 (79.7) | 1705 (5.8) | 2787 (9.5) | 29,413 (100) |
| IGA, n (%) | 24,926 (84.7) | 1706 (5.8) | 2788 (9.5) | 29,420 (100) |

AD, atopic dermatitis; EASI, Eczema Area and Severity Index; EOS, eosinophil count; E-R, exposure–response; IGA, Investigator’s Global Assessment; N, number of patients; SD, standard deviation; TARC, thymus and activation-regulated chemokine; TCS, topical corticosteroids.

**Fig. S1** Diagnostic plots for EASI score final model showing A) transformed observed vs IPRED and observed vs back-transformed IPRED, B) IWRES, C) IWRES after time from first dose, D) goodness-of-fit plots, and E) empirical Bayes predictions of random baseline and placebo effects.

**A.**


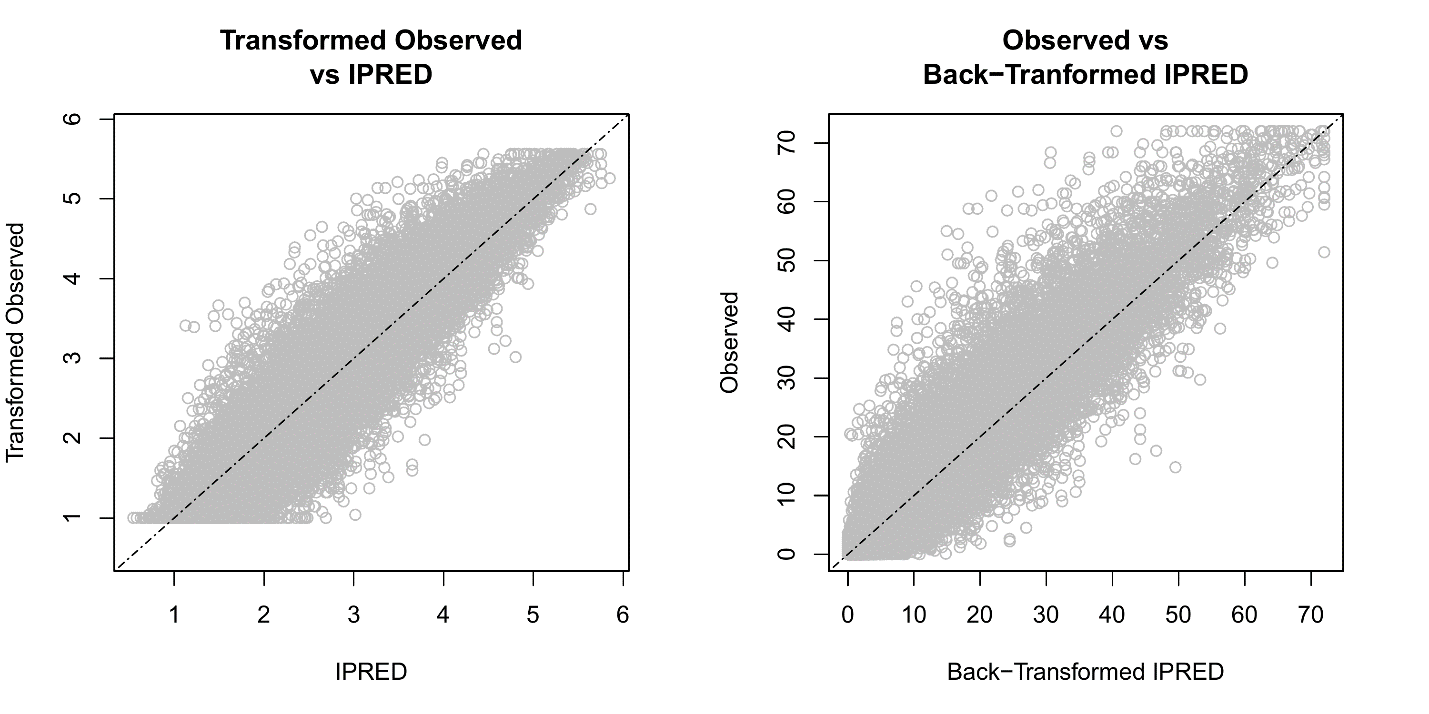


**B.**


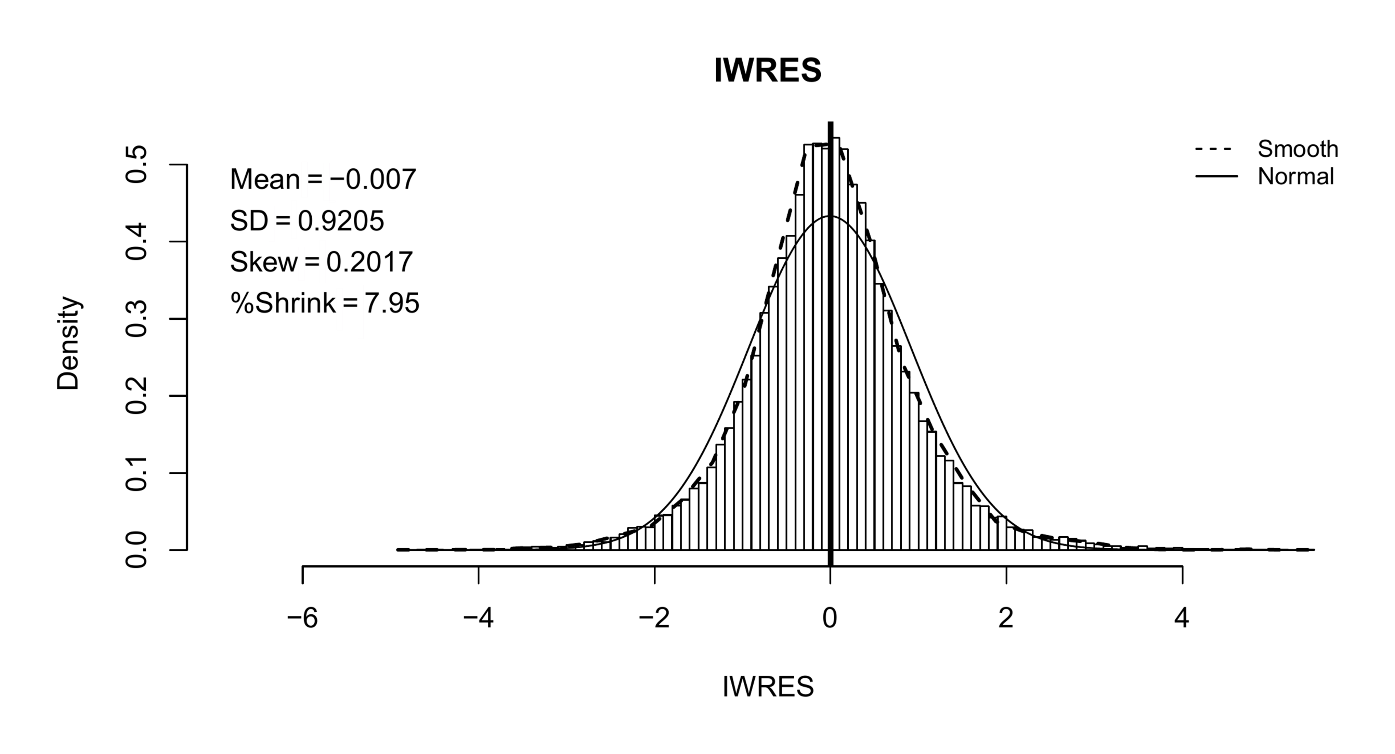


**C.**


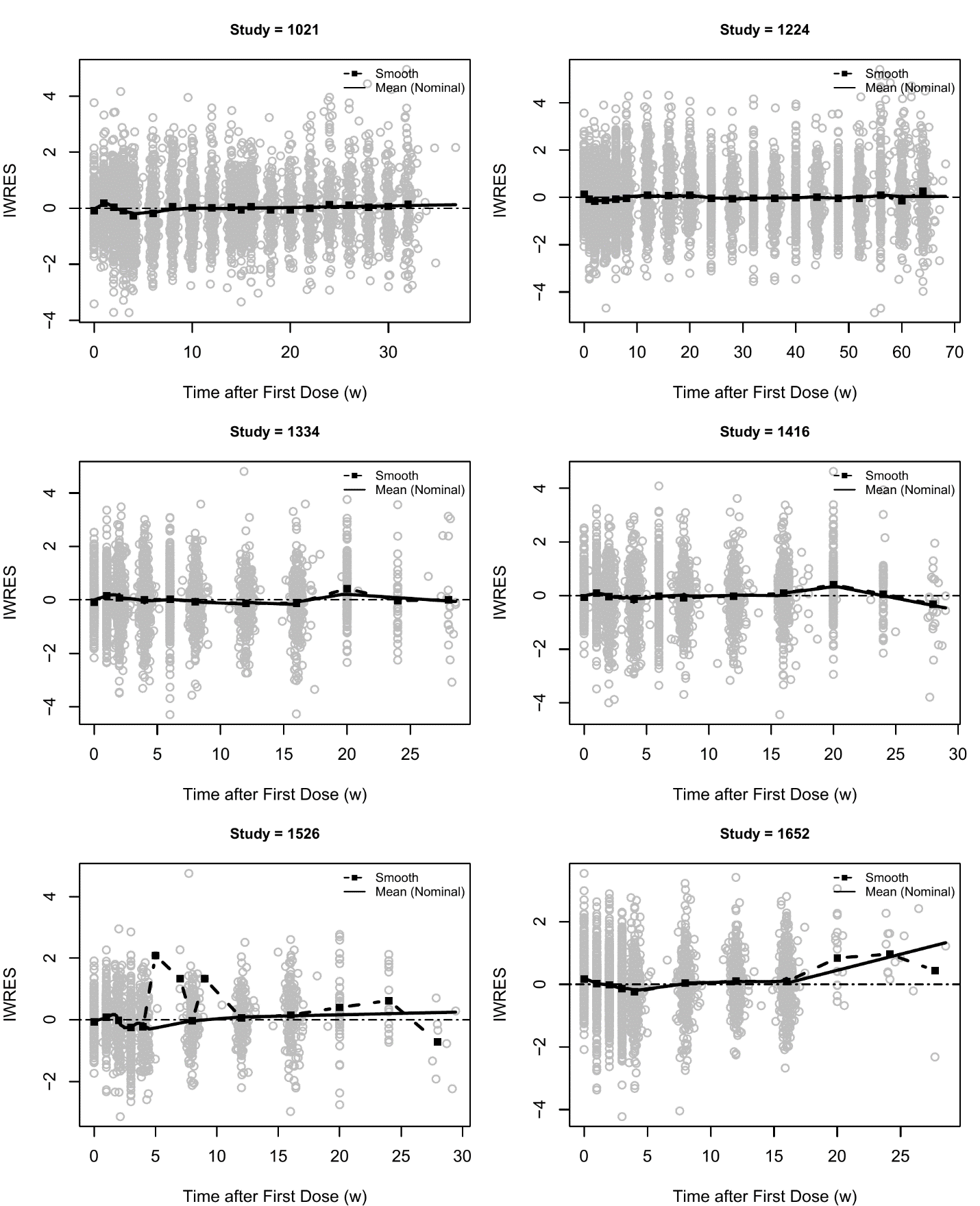


**D.**


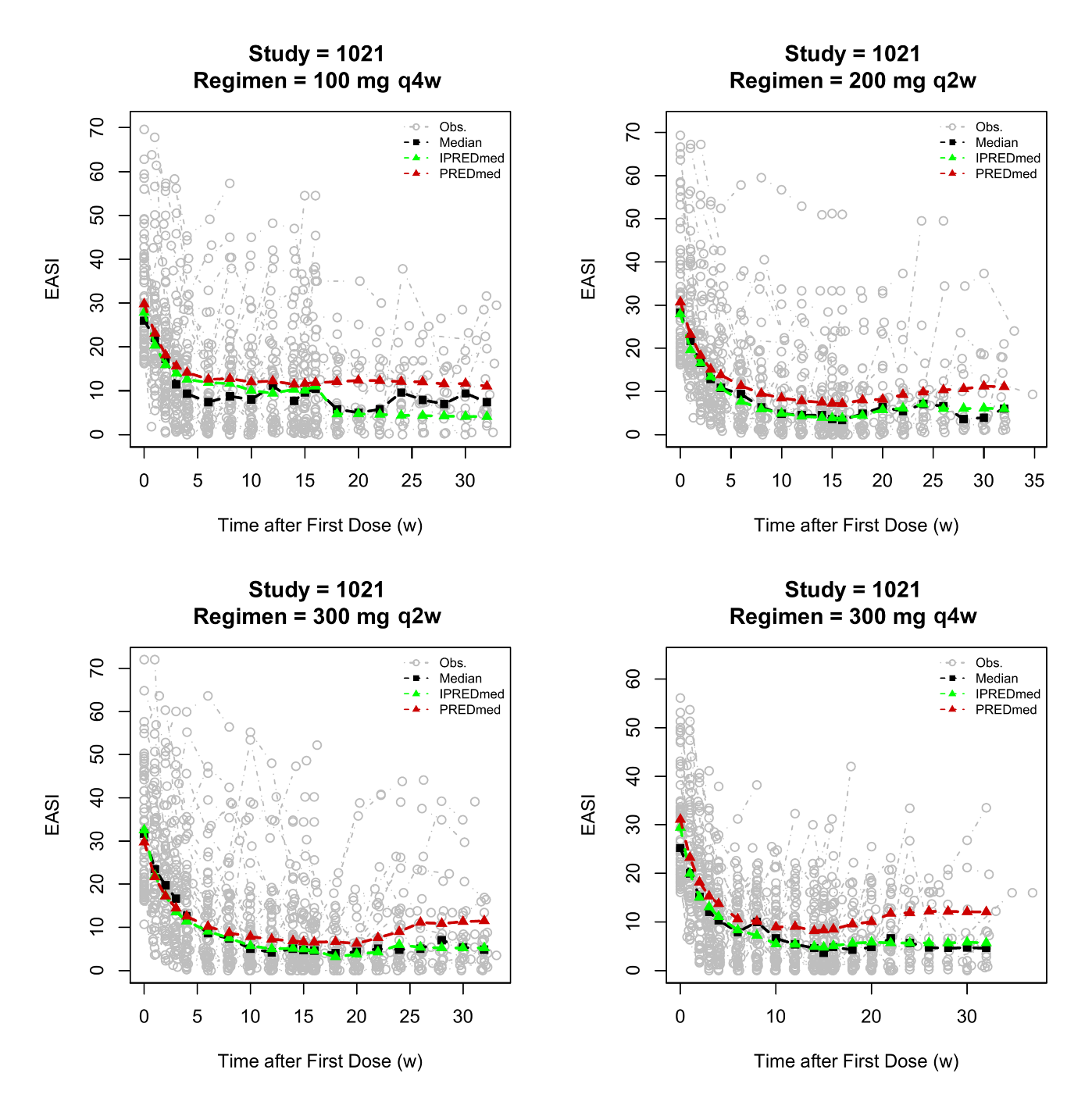


**E.**


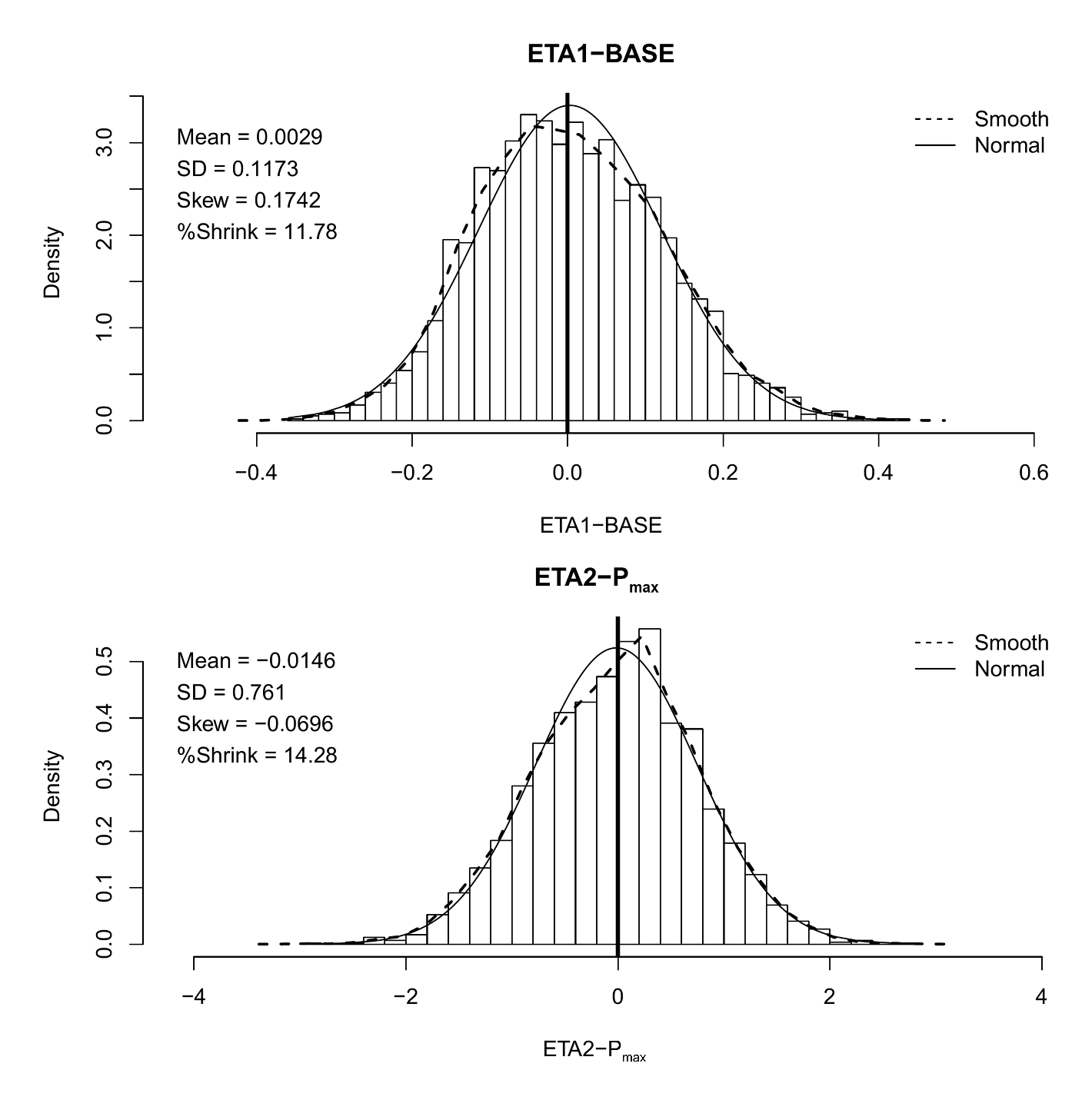


BASE, baseline; EASI, Eczema Area and Severity Index; ETA, empirical Bayes prediction of the interindividual random effect in a PK or PD parameter; IPRED, individual predicted value based on individual’s ETA; IWRES, individual weighted residuals; PD, pharmacodynamic; PK, pharmacokinetic; P_max_, maximum placebo effect; q2w, every 2 weeks; q4w, every 4 weeks; SD, standard deviation; w, weeks.

**Fig. S2** Goodness-of-fit of the dupilumab IGA final model (A) by study and ETA overall (B) and by age group (C).

**A.**

**
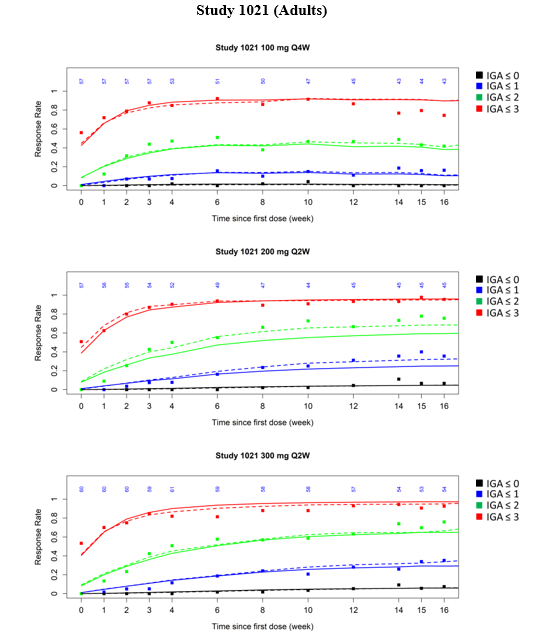
**

**Study 1021 (Adults)**


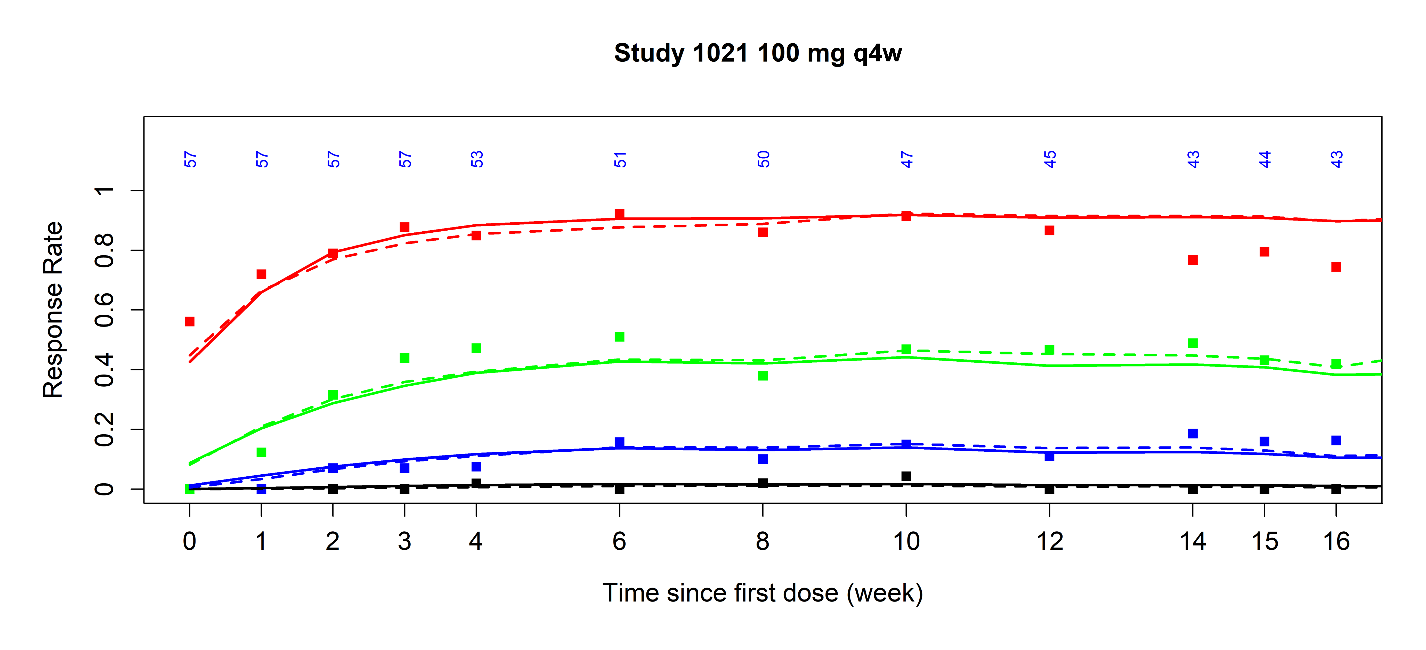


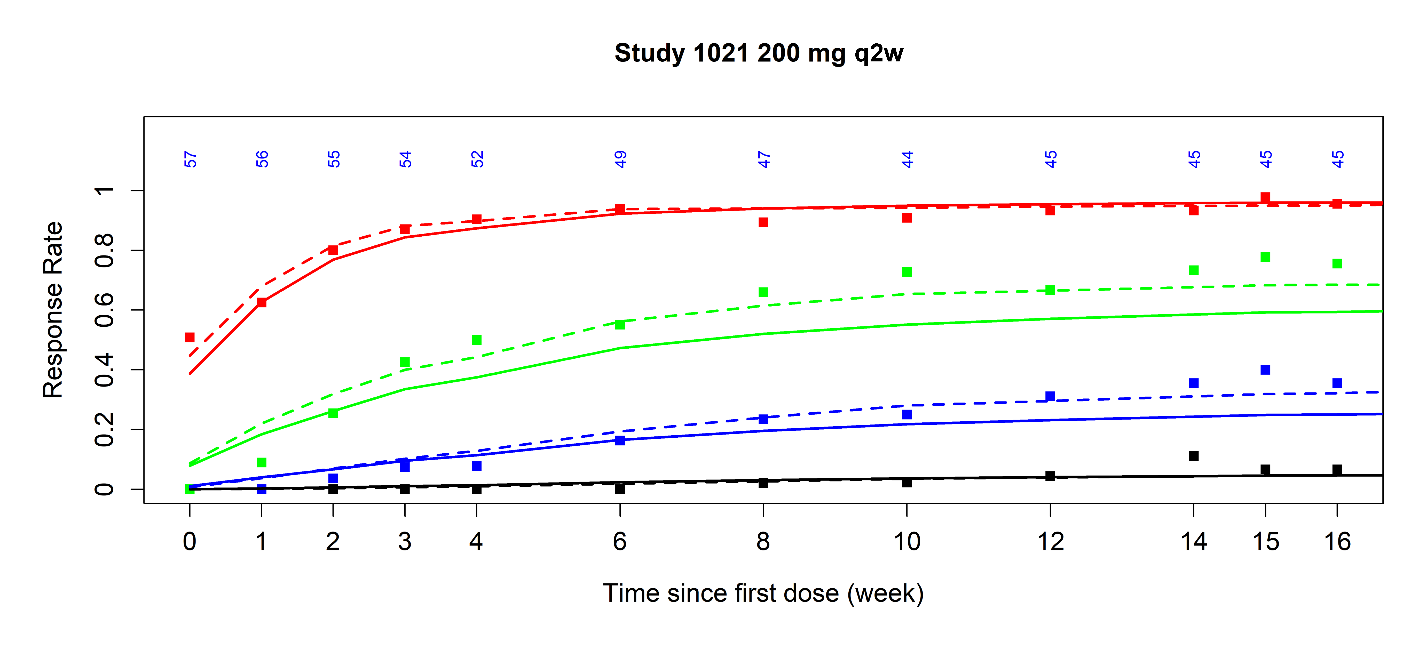


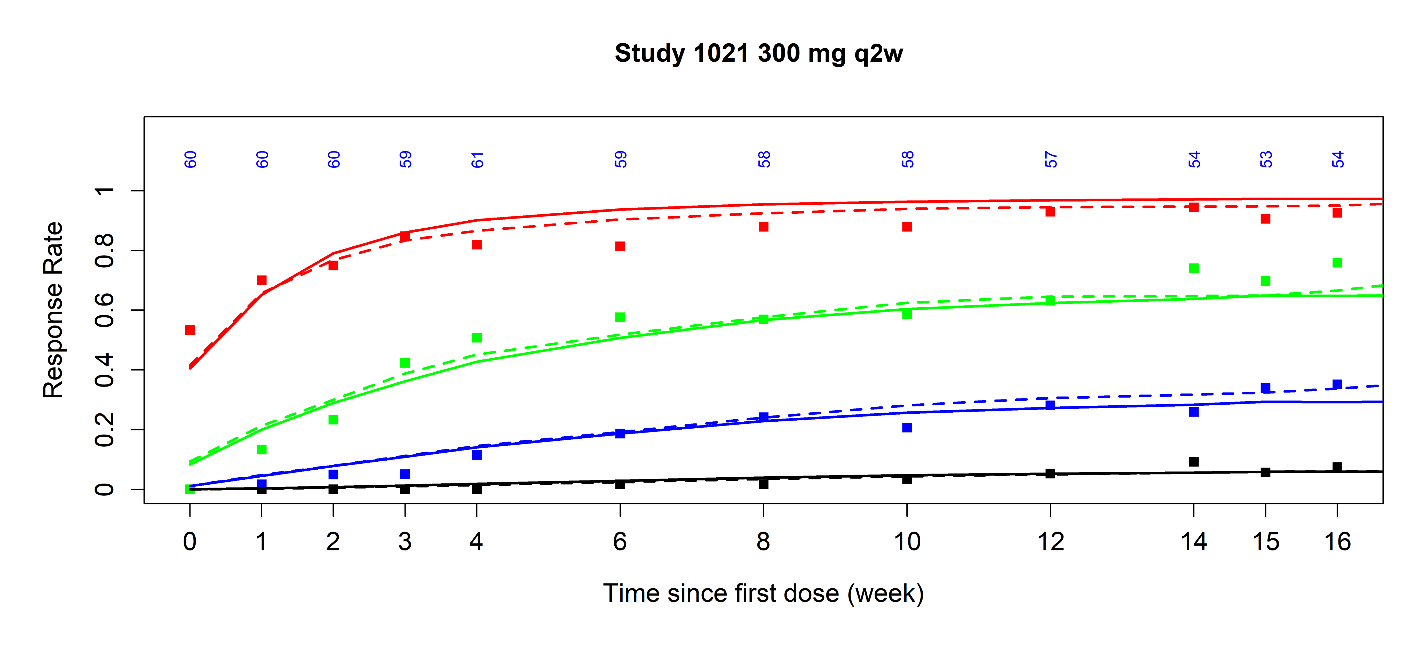


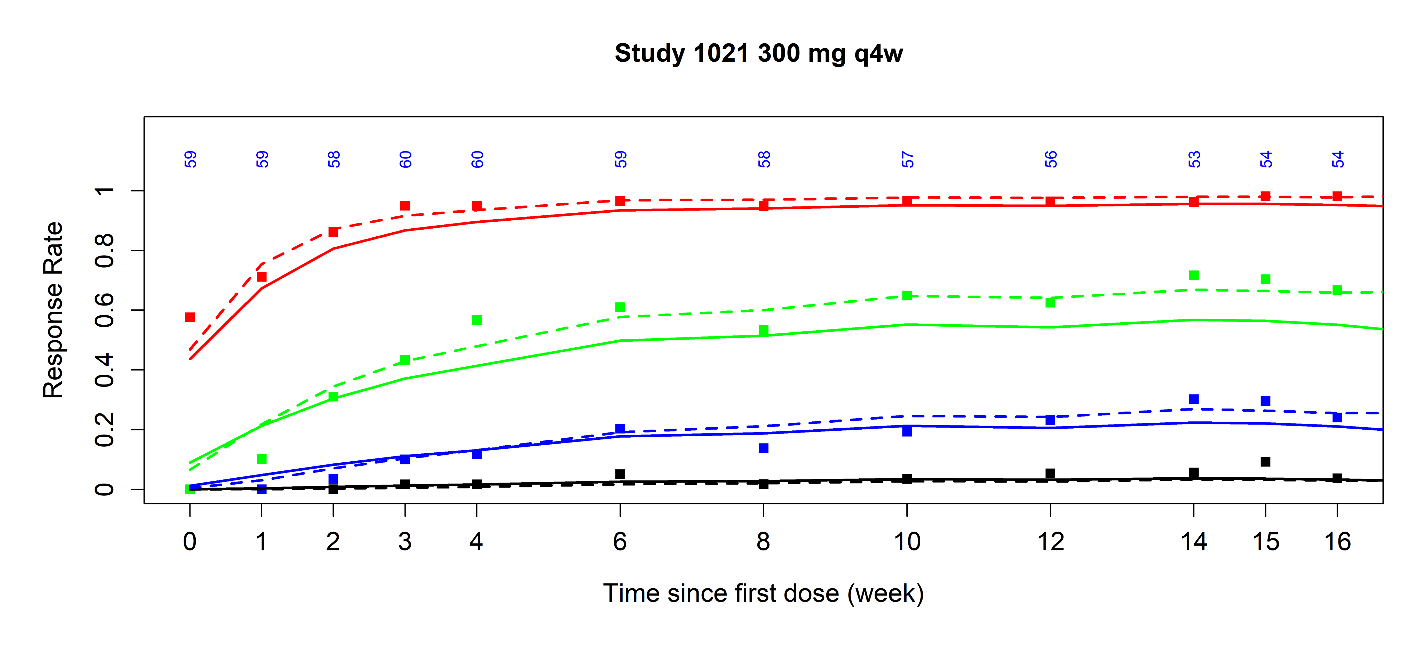


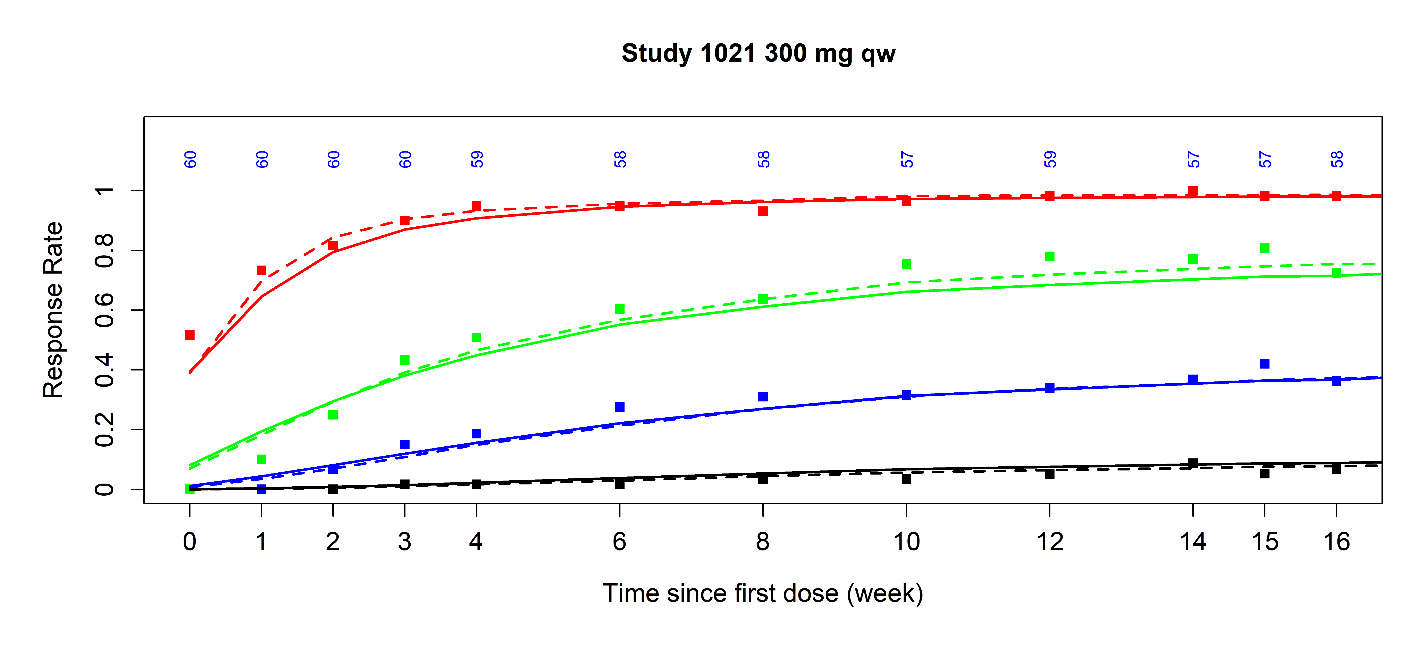


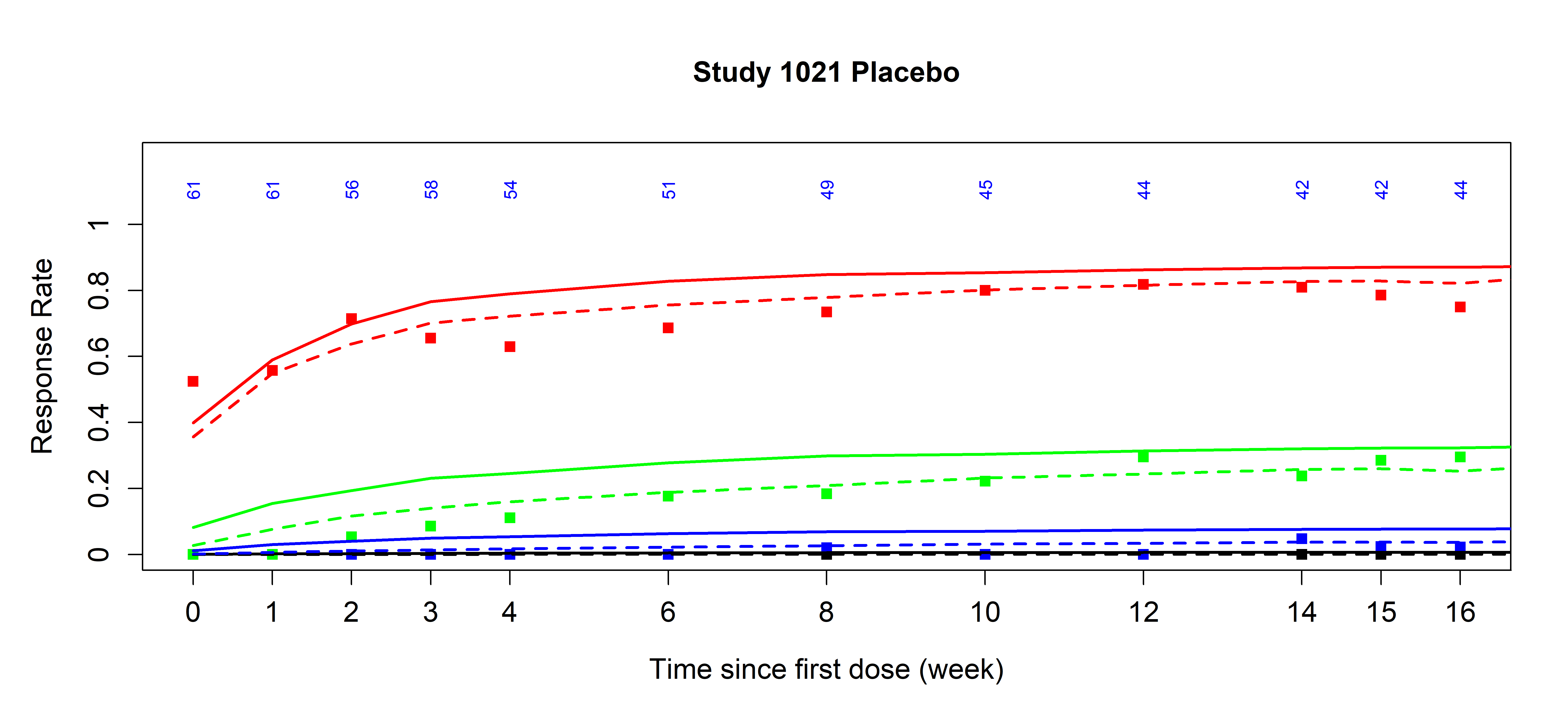


**Study 1224 (Adults)**


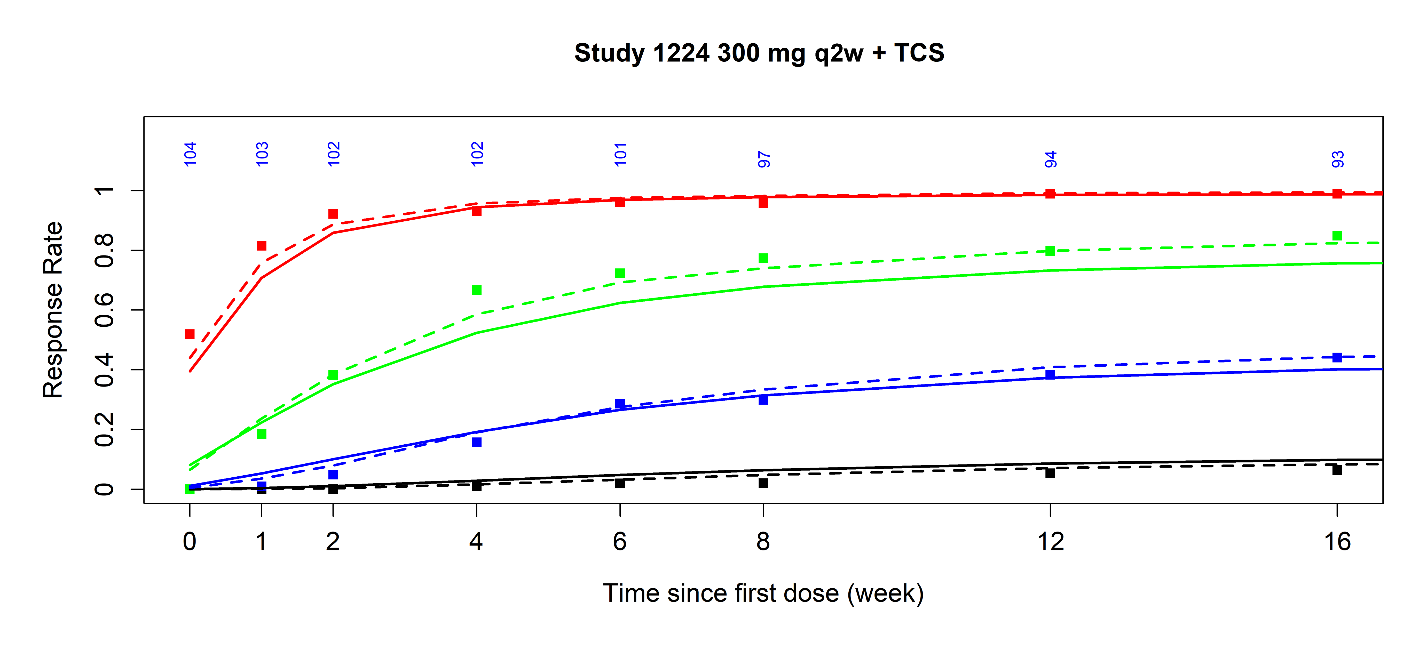


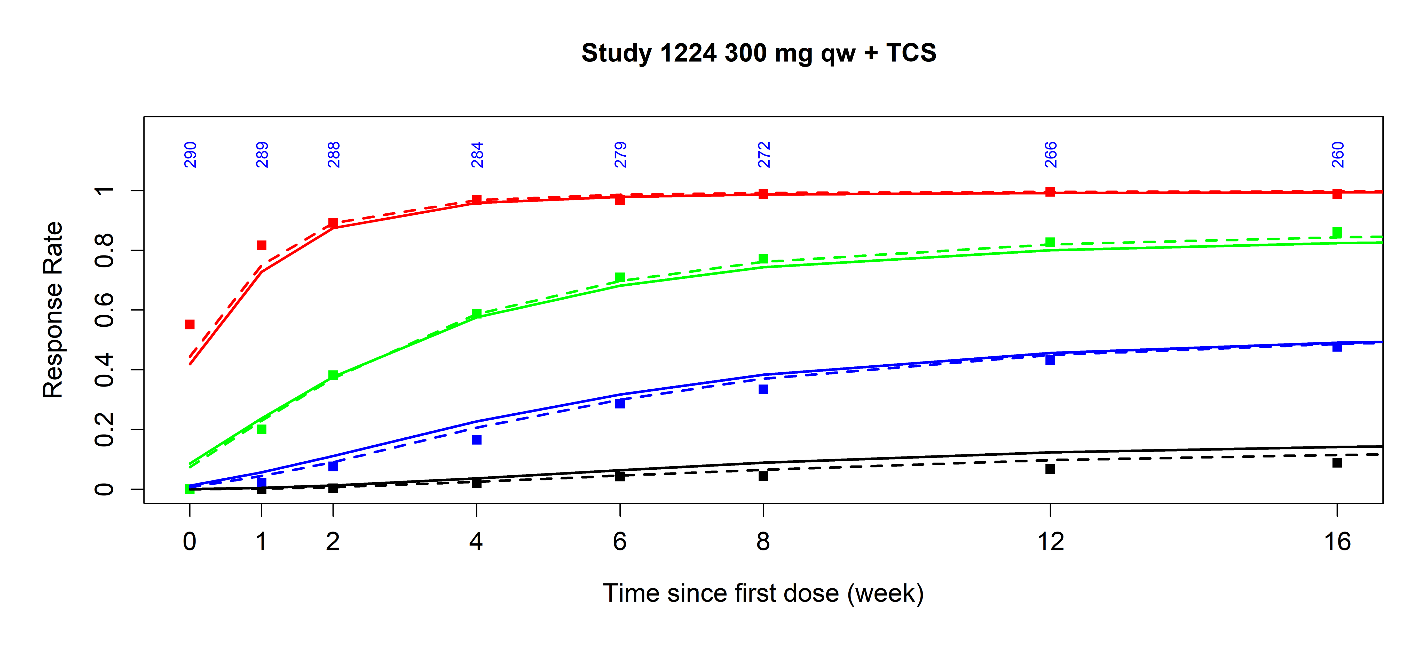


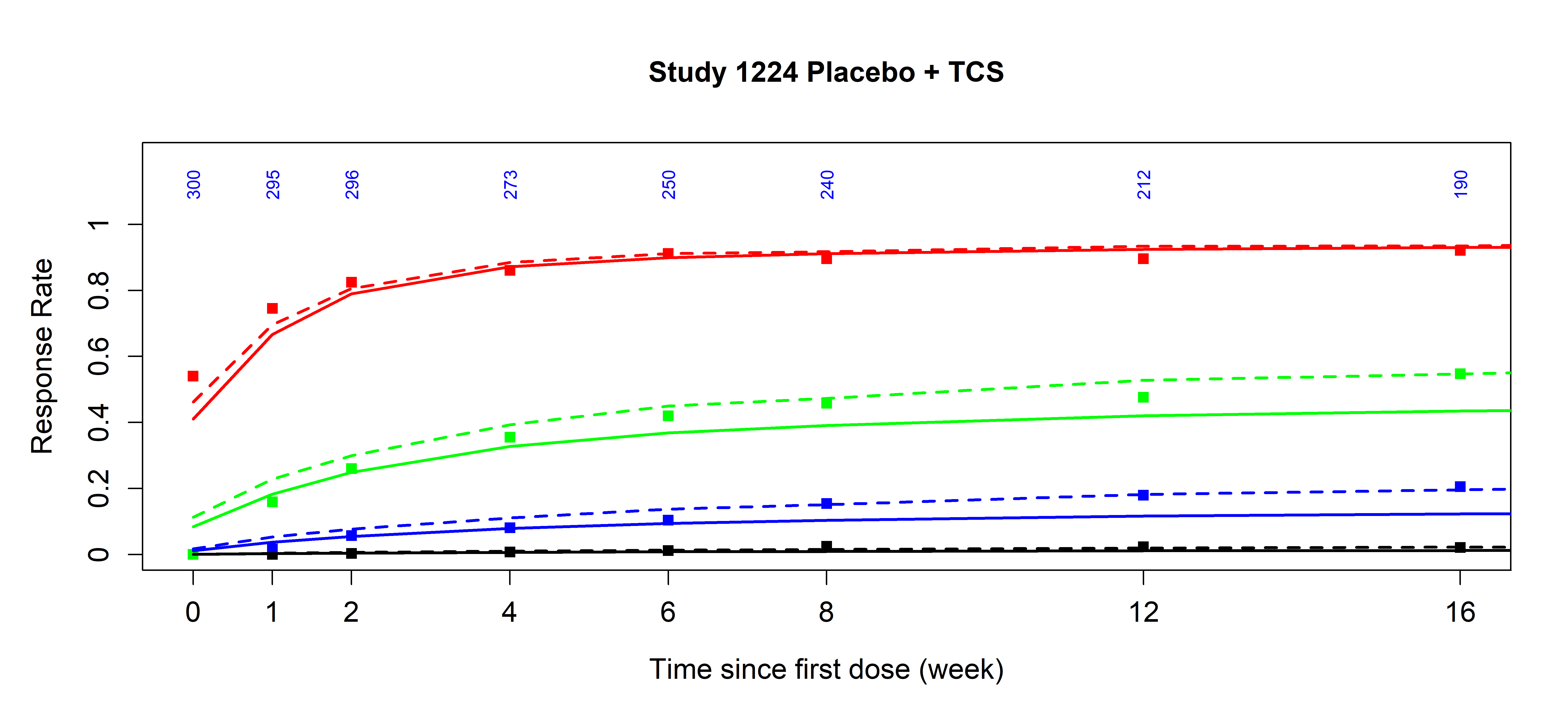


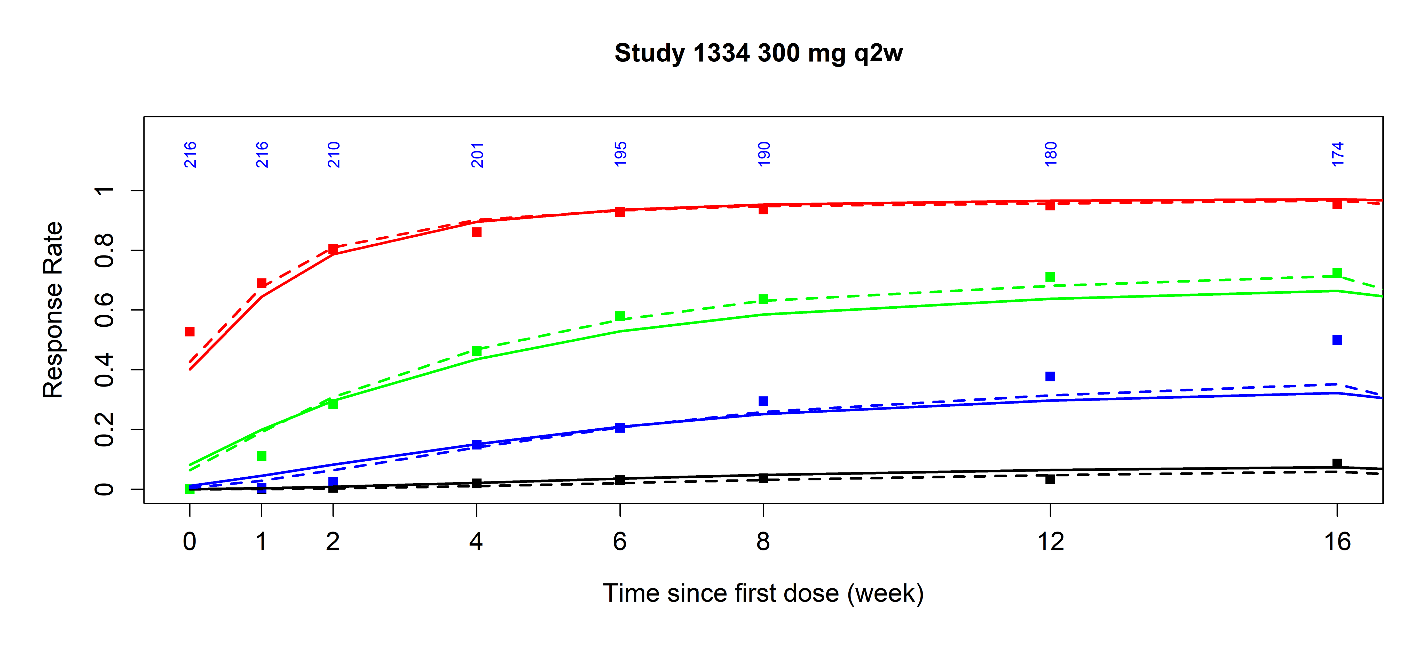


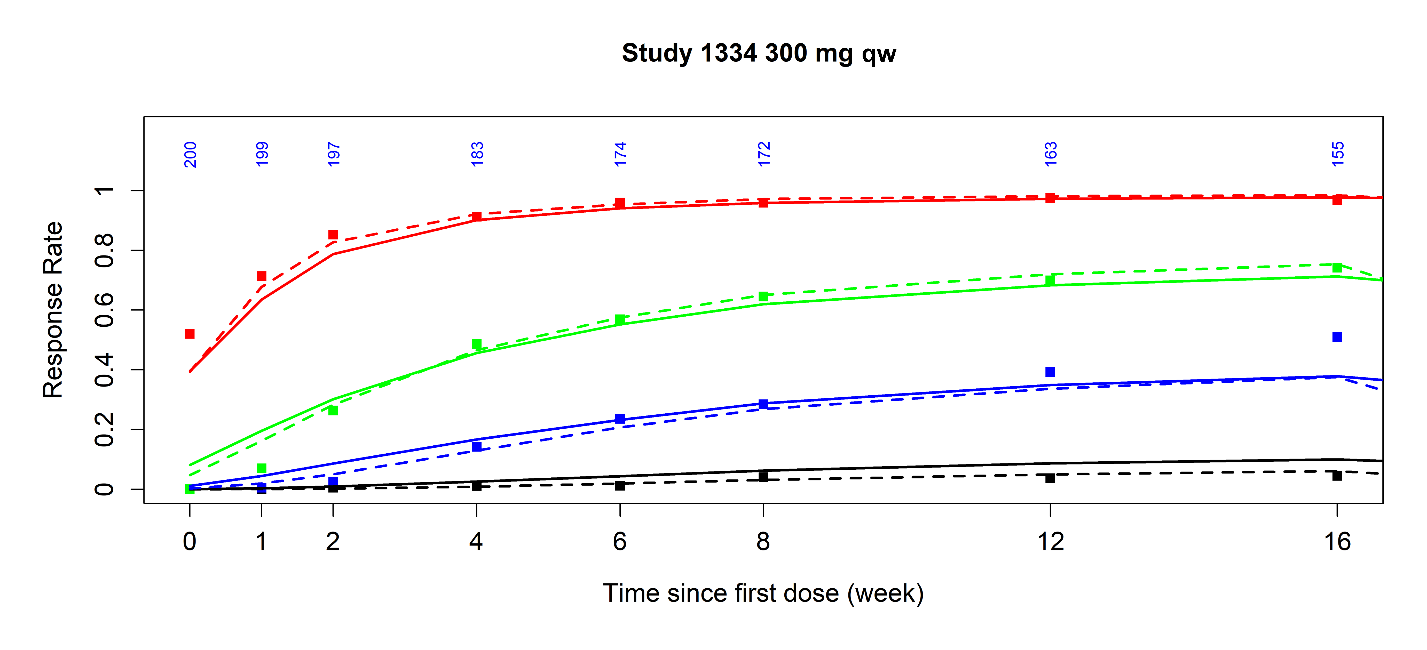


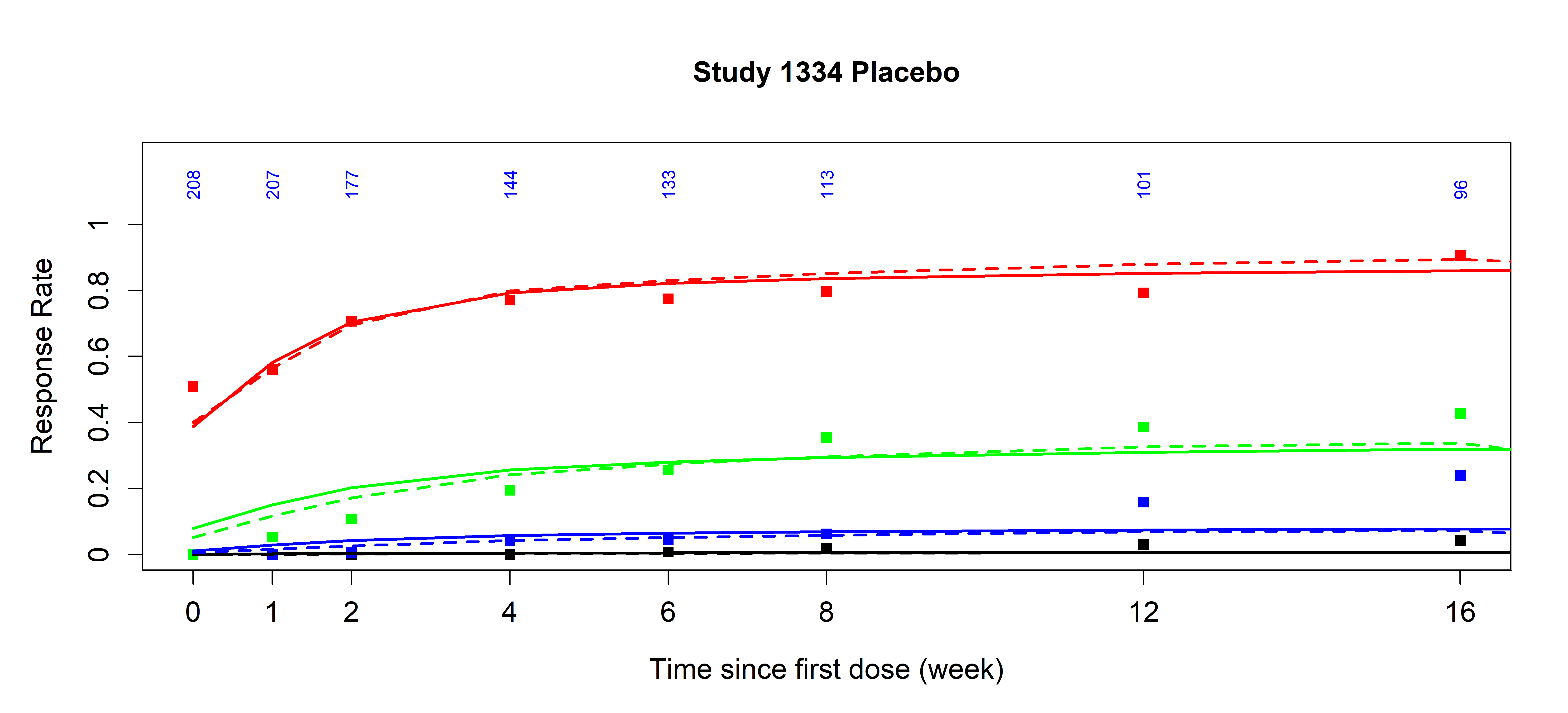


**Study 1416 (Adults)**


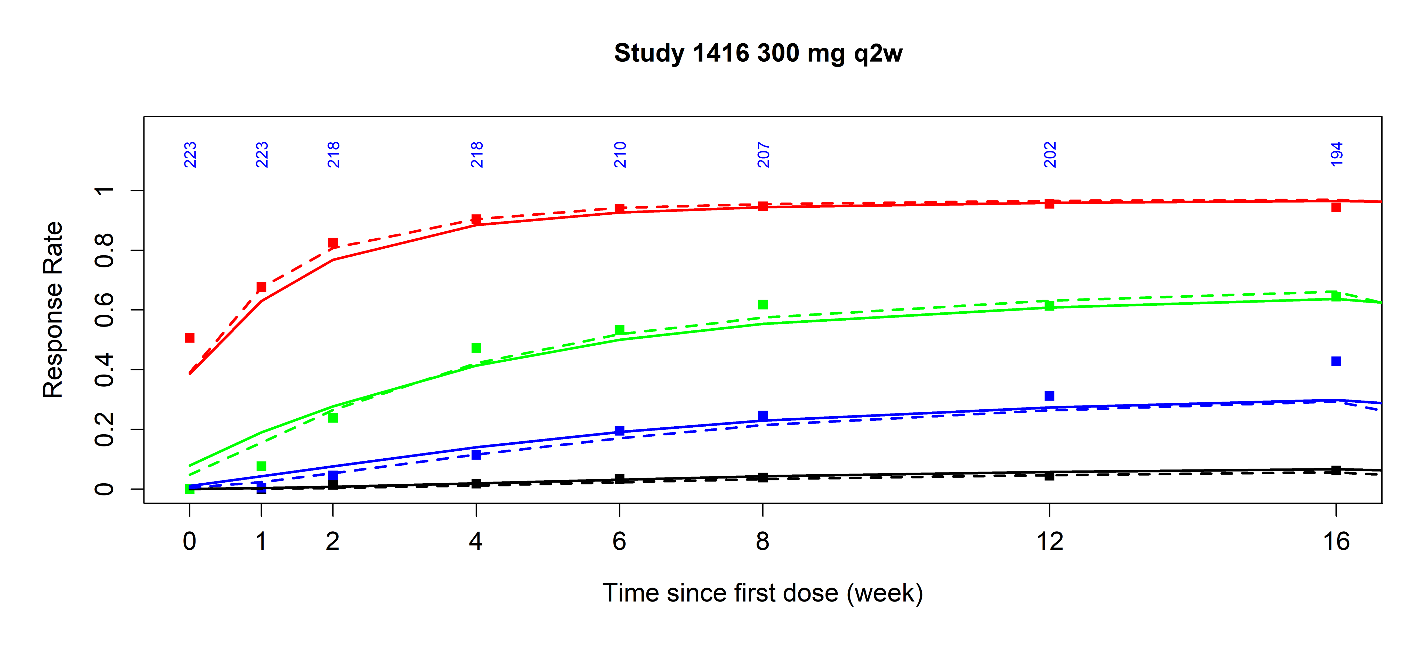


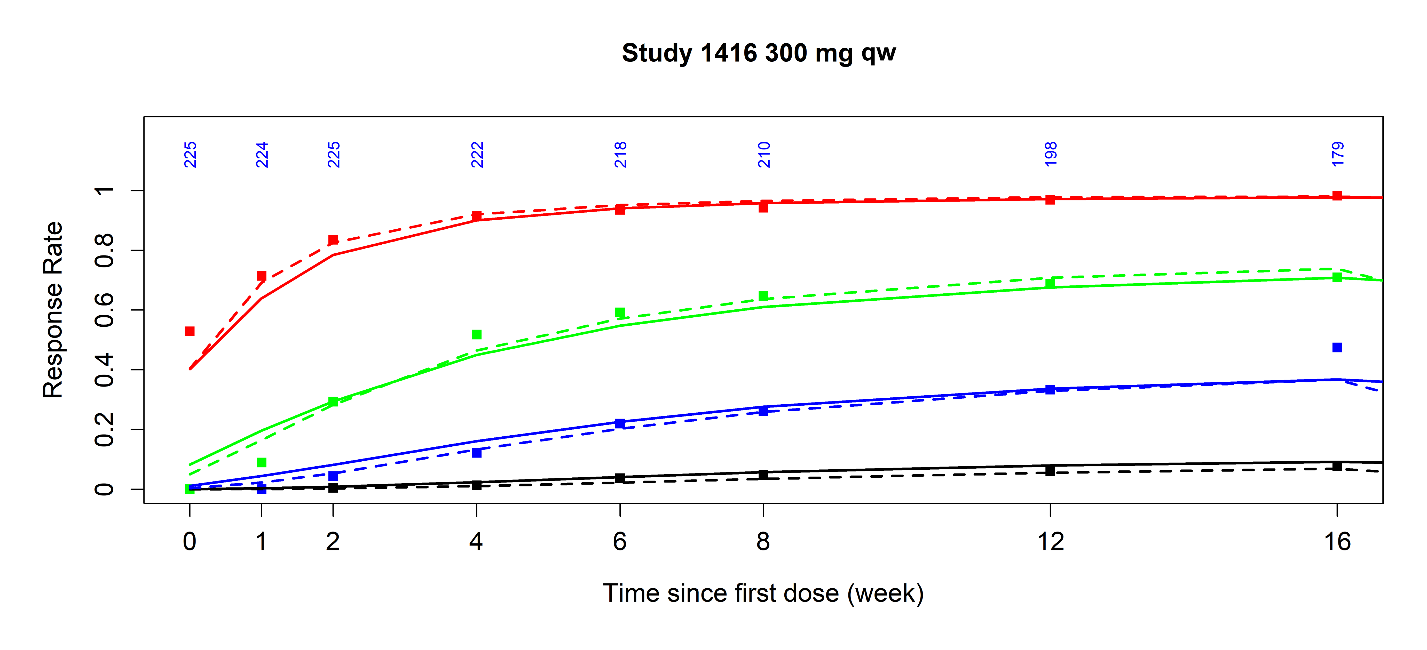


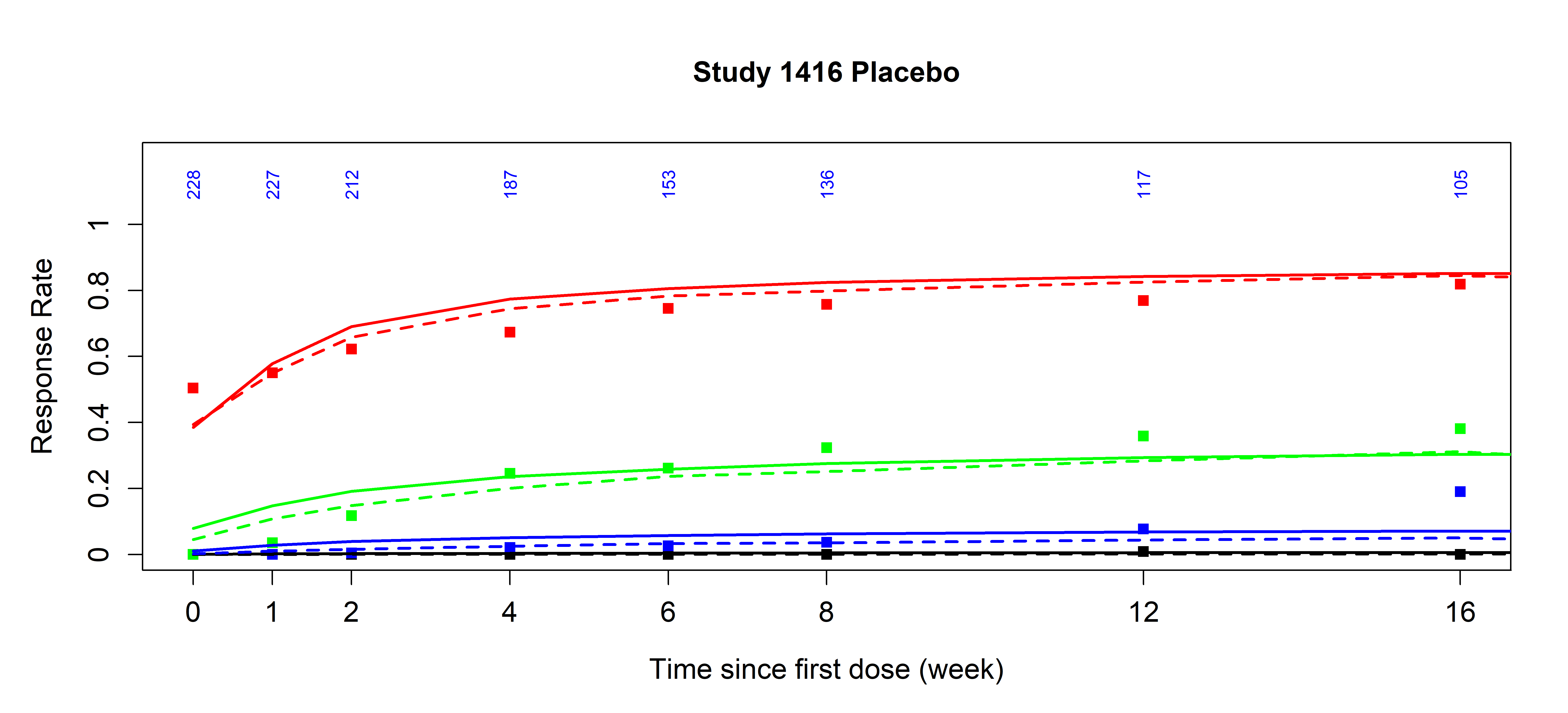


**Study 1526 (Adolescents)**


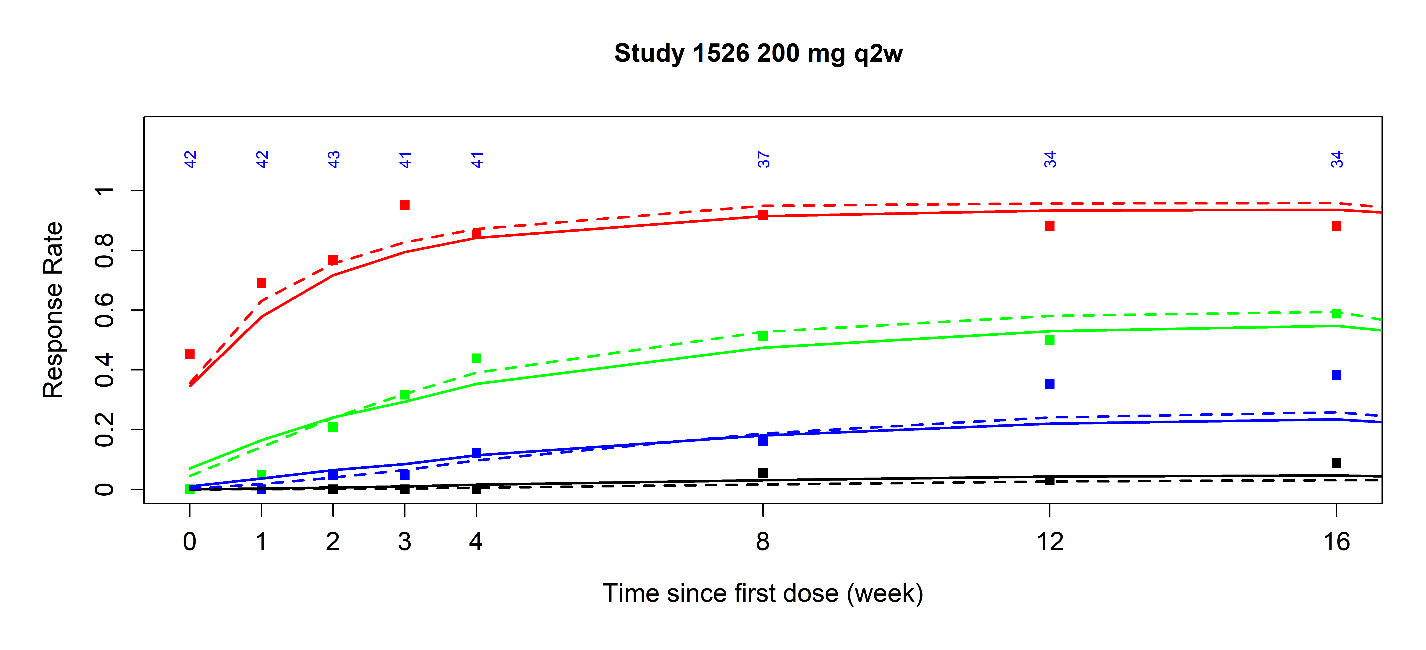


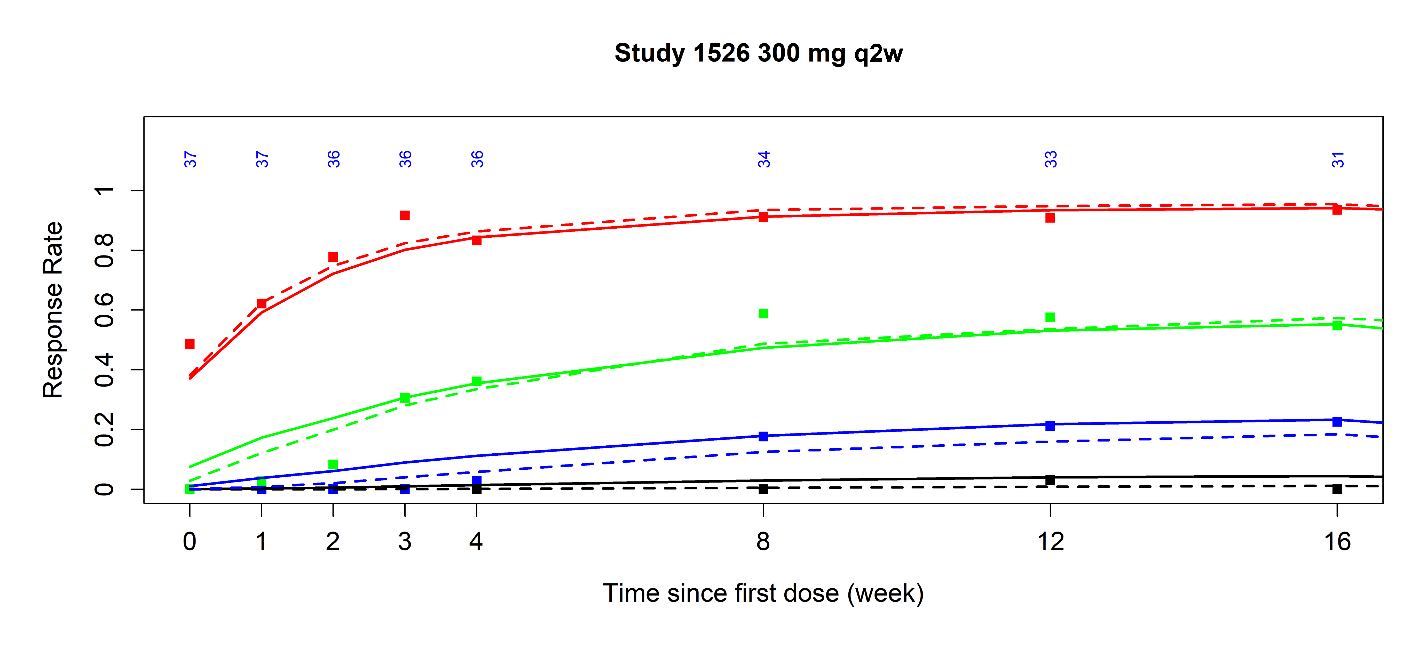


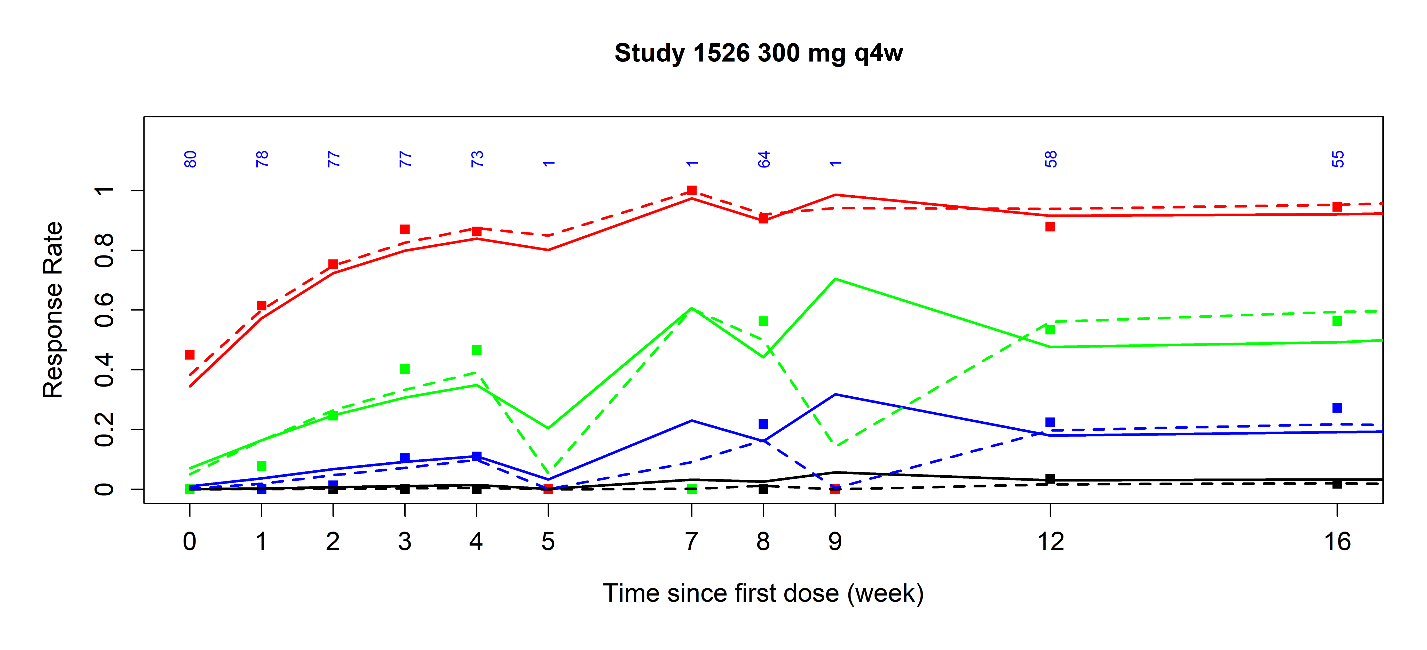


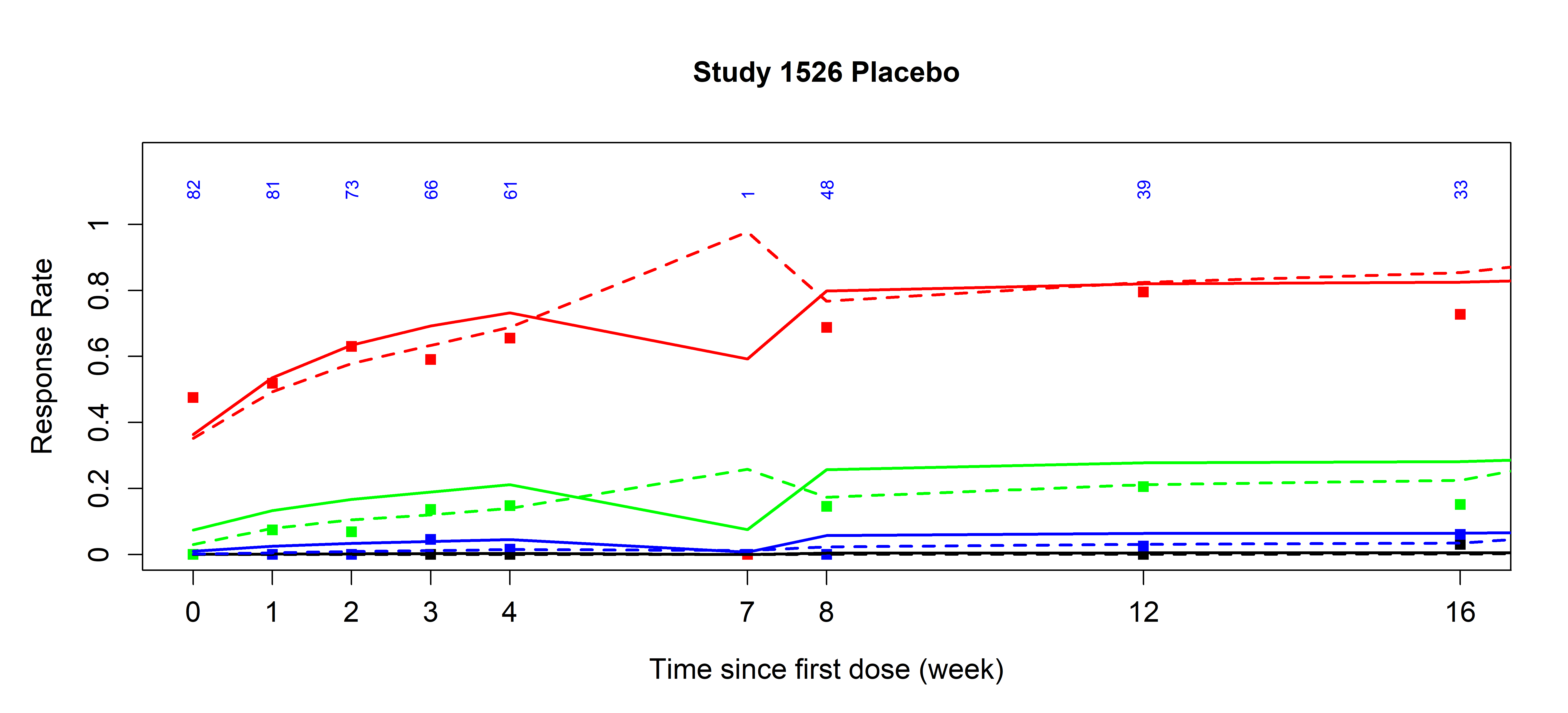


**Study 1652 (Children)**


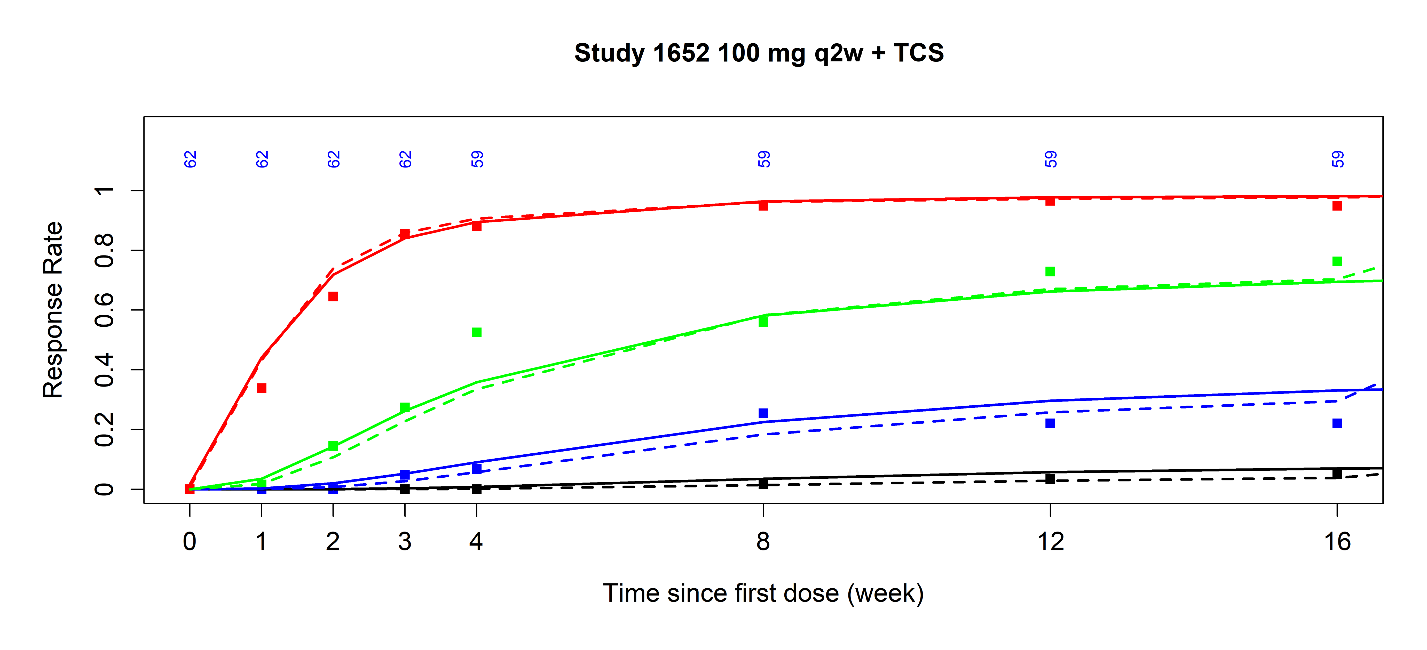


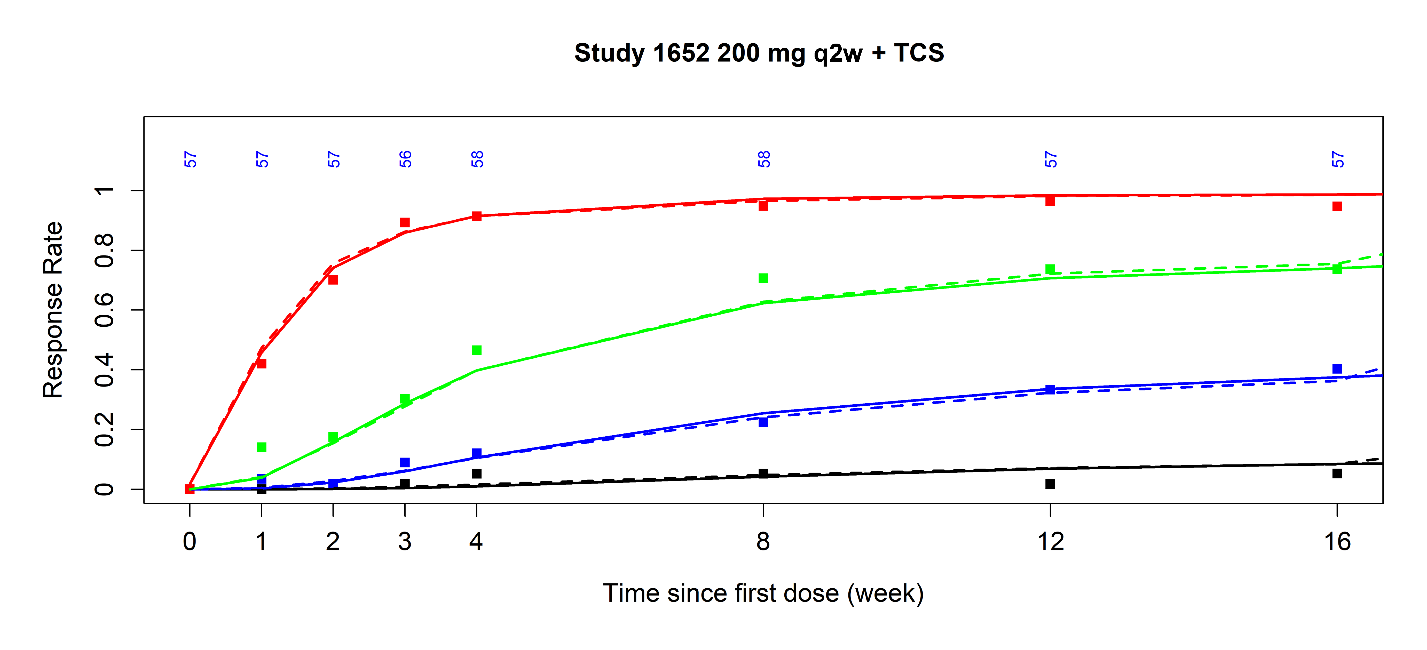


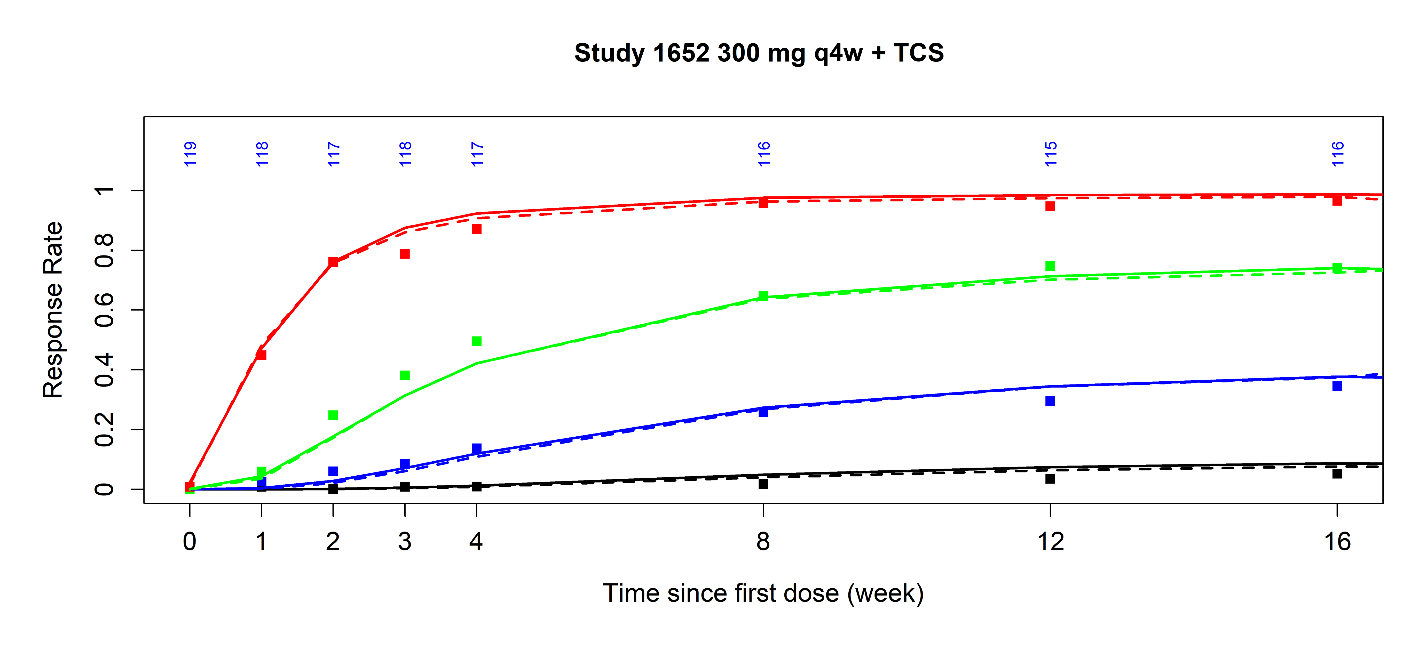


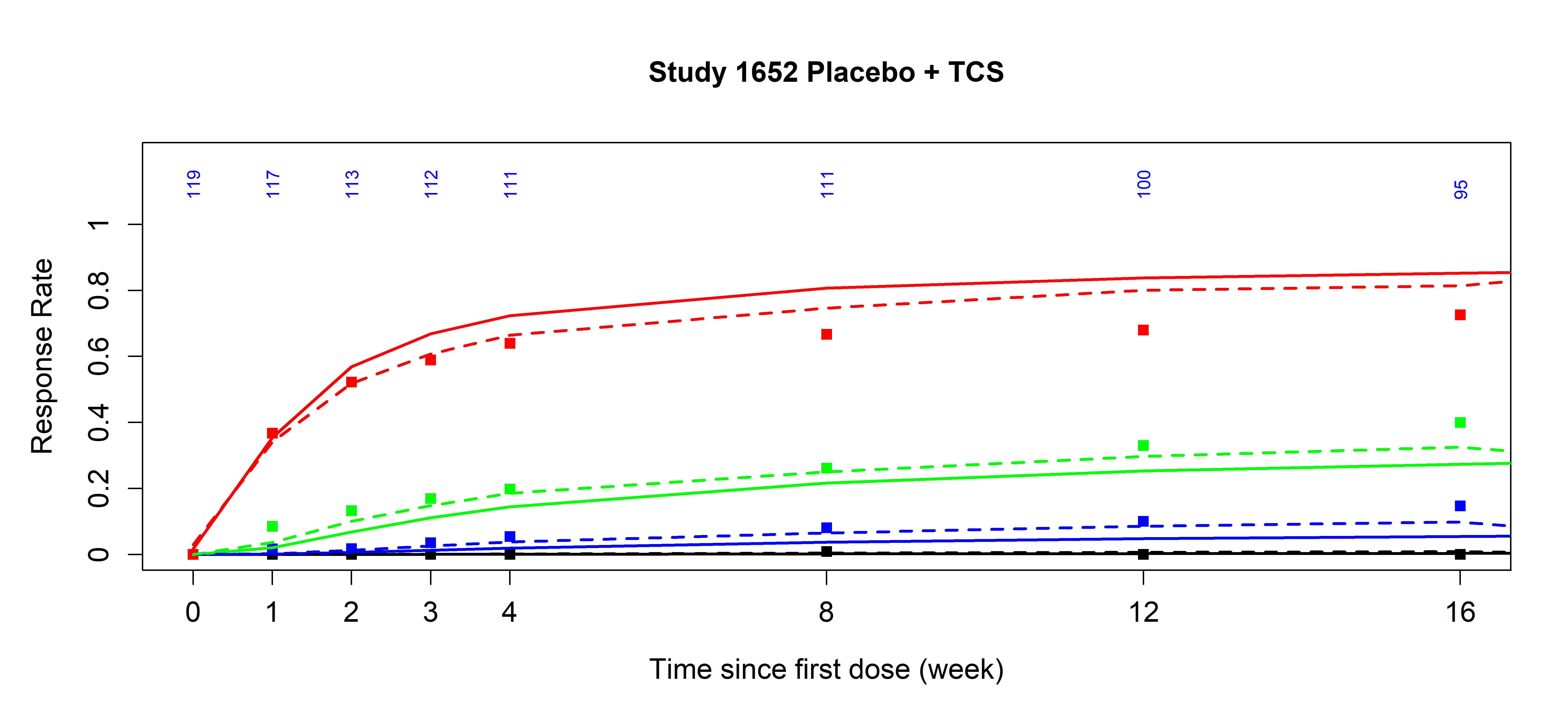


**B.**


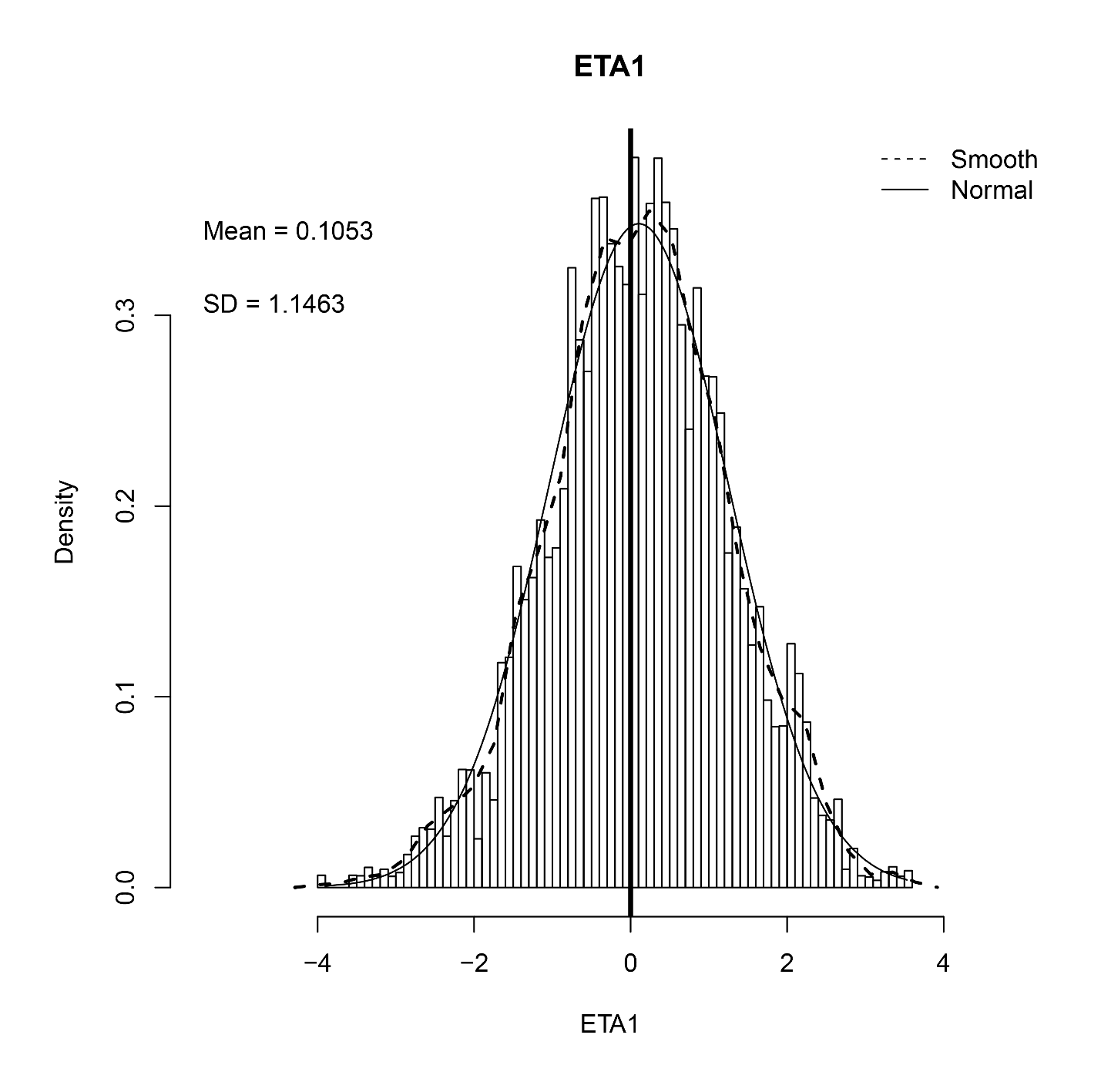


**C.**


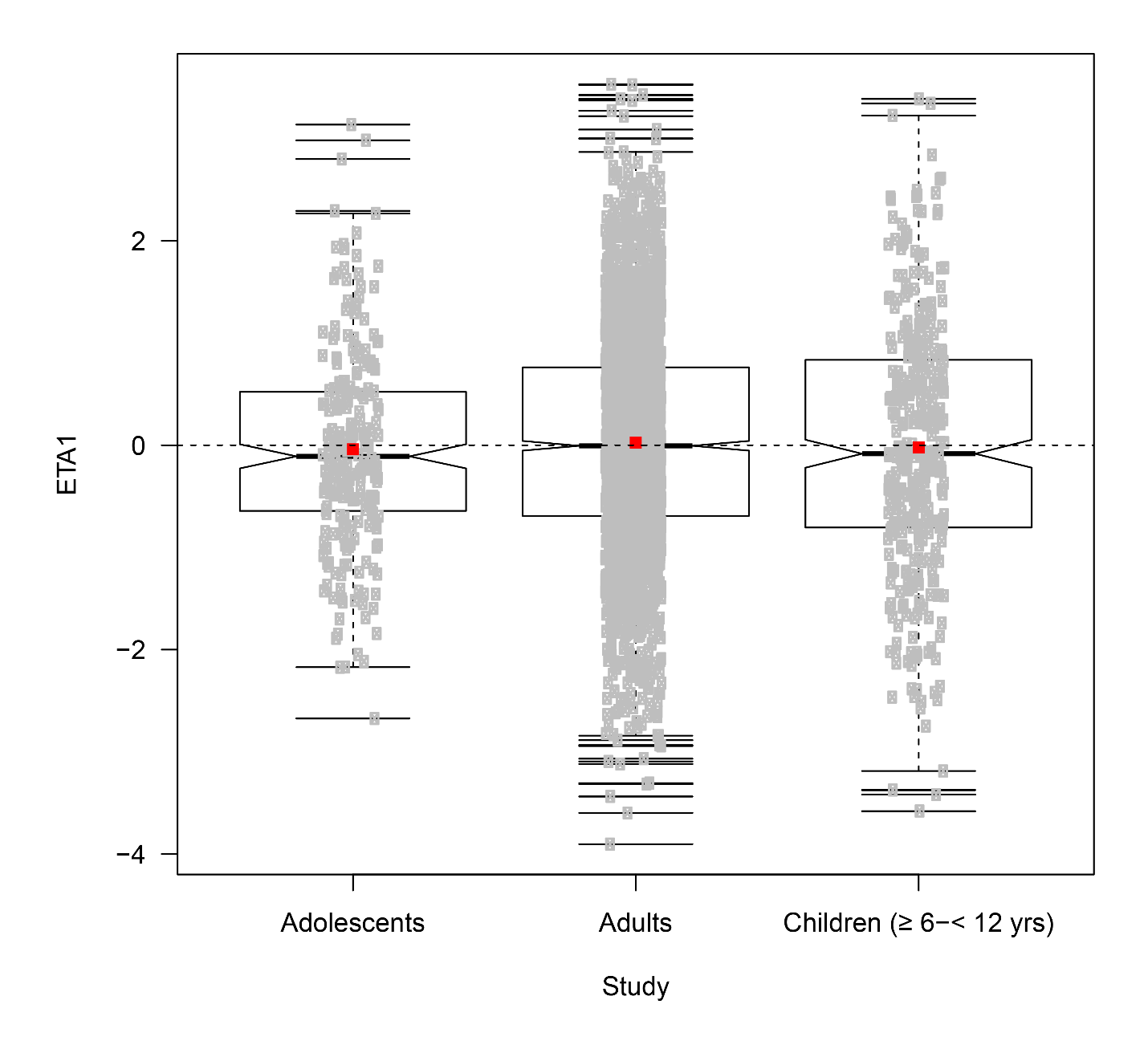


Panel A: Black, blue, green, and red solid squares correspond to observed responder rates for IGA ≤ 0, IGA ≤ 1, IGA ≤ 2, and IGA ≤ 3, respectively. Solid and dashed lines (black, blue, green, and red) represent corresponding population mean predictions and averages of IPREDs of cumulative response rates for IGA ≤ 0, IGA ≤ 1, IGA ≤ 2, and IGA ≤ 3, respectively. The number of observations used in calculation of mean values are shown in blue.

ETA, empirical Bayes prediction of the interindividual random effect in a PK or PD parameter**;** IGA, Investigator’s Global Assessment; IPRED, individual predicted value based on individual’s ETAs; PD, pharmacodynamic; PK, pharmacokinetic; q2w, every 2 weeks; q4w, every 4 weeks; qw, every week; SD, standard deviation; TCS, topical corticosteroids.

**Fig. S3** Model-predicted E-R profiles at Week 16 for dupilumab without TCS in patients with severe disease by age group.

**
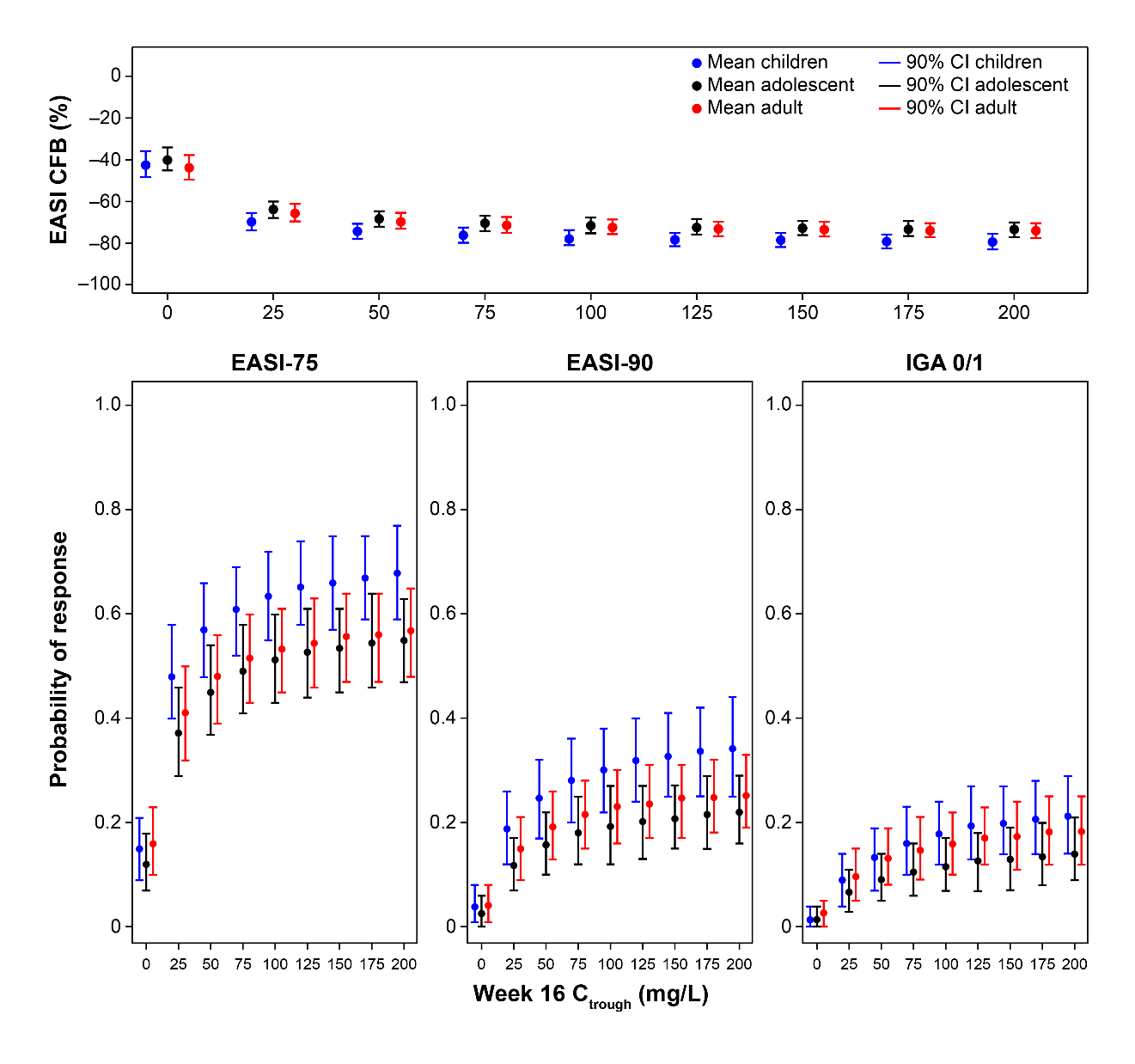
**

CI, confidence interval; CFB, change from baseline; C_trough_, dupilumab concentration at the end of a dosing interval; EASI-75/90, ≥ 75%/90% improvement from baseline in Eczema Area and Severity Index scores; E-R, exposure–response; IGA, Investigator’s Global Assessment; TCS, topical corticosteroids.

**Fig. S4** Model-predicted E-R profiles at Week 16 for dupilumab with TCS in patients with moderate disease by age group.


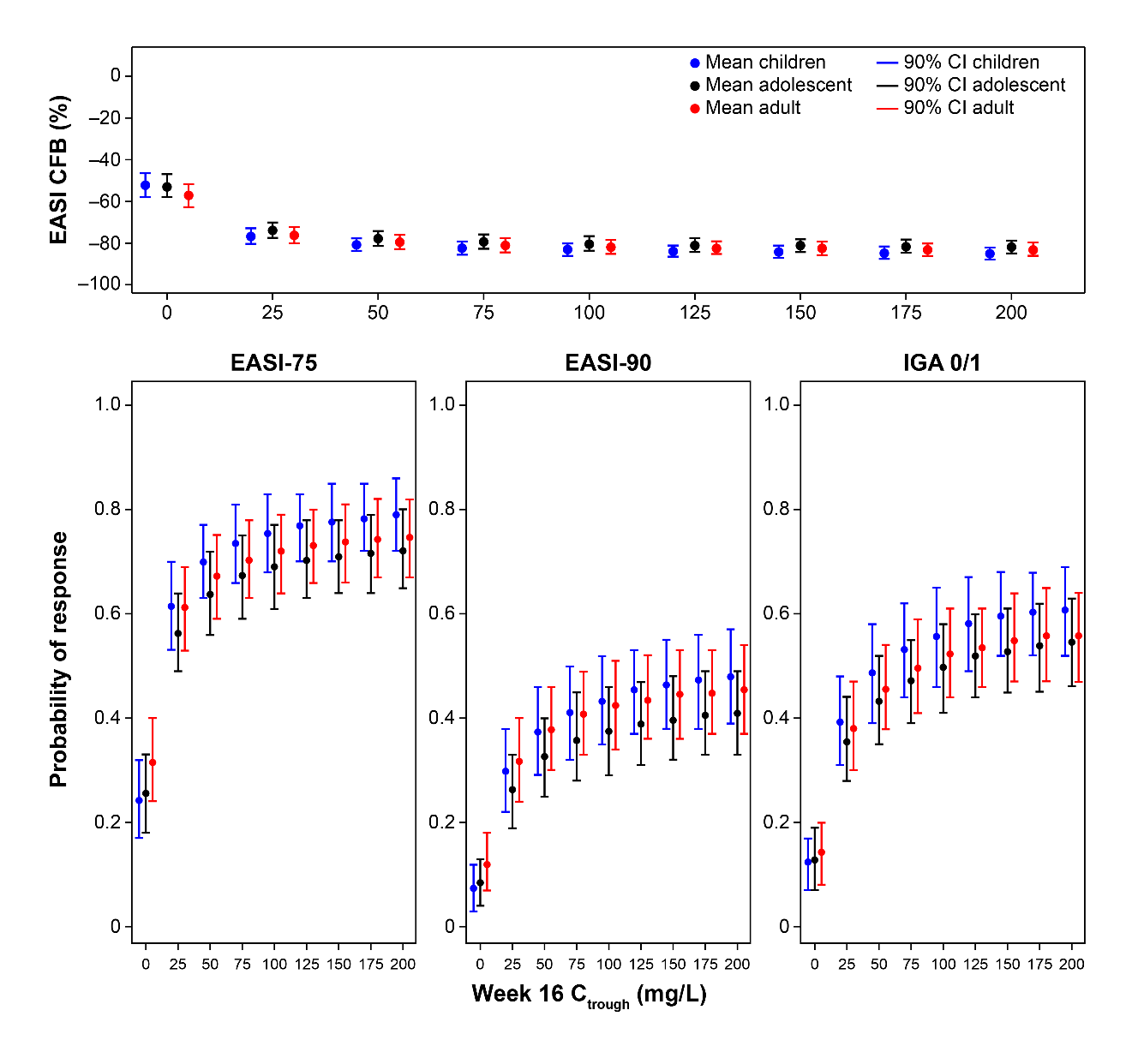


CI, confidence interval; CFB, change from baseline; C_trough_, dupilumab concentration at the end of a dosing interval; EASI-75/90, ≥ 75%/90% improvement from baseline in Eczema Area and Severity Index scores; E-R, exposure–response; IGA, Investigator’s Global Assessment; TCS, topical corticosteroids.

**Fig. S5** Model-predicted E-R profiles at Week 16 for dupilumab without TCS in patients with moderate disease by age group.

**
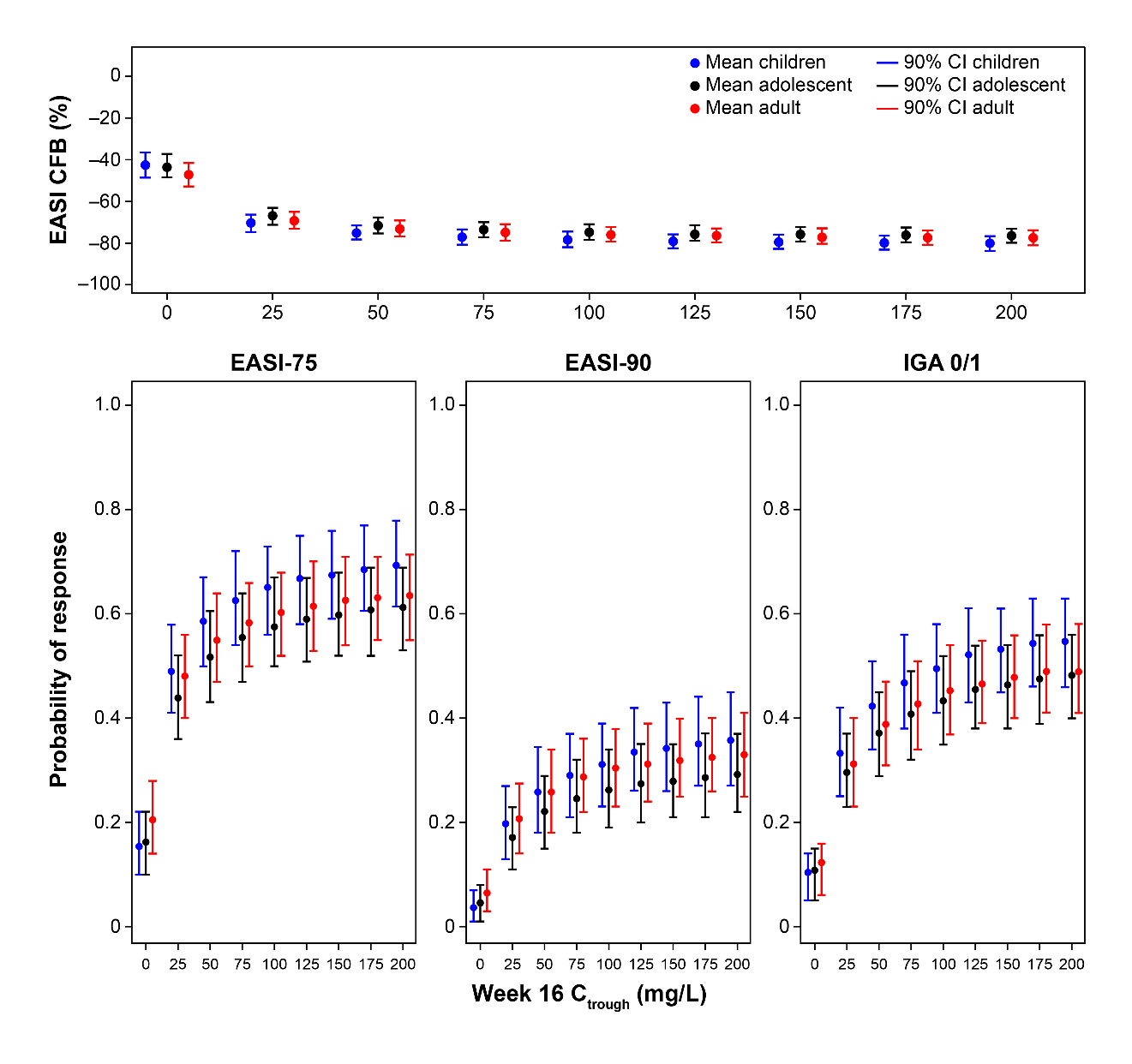
**

CI, confidence interval; CFB, change from baseline; C_trough_, dupilumab concentration at the end of a dosing interval; EASI-75/90, ≥ 75%/90% improvement from baseline in Eczema Area and Severity Index scores; E-R, exposure–response; IGA, Investigator’s Global Assessment; TCS, topical corticosteroids.

**References**

1. Hanifin JM, Thurston M, Omoto M, Cherill R, Tofte SJ, Graeber M. The eczema area and severity index (EASI): assessment of reliability in atopic dermatitis. EASI Evaluator Group. Exp Dermatol. 2001;10(1):11–18.
2. Eczema Council. Validated Investigator Global Assessment scale for Atopic Dermatitis vIGA-AD™. <https://www.eczemacouncil.org/assets/docs/Validated-Investigator-Global-Assessment-Scale_vIGA-AD_2017.pdf>. Accessed 13 October 2023.
3. Sharma A, Jusko WJ. Characteristics of indirect pharmacodynamic models and applications to clinical drug responses. Br J Clin Pharmacol. 1998;45(3):229–39.
4. Hutmacher MH, Krishnaswami S, Kowalski KG. Exposure-response modeling using latent variables for the efficacy of a JAK3 inhibitor administered to rheumatoid arthritis patients. J Pharmacokinet Pharmacodyn. 2008;35(2):139–57.
5. Montgomery DC, Peck EA, Vining GG. Introduction to linear regression analysis. Hoboken, NJ, USA: John Wiley & Sons; 2012.
6. Kovalenko P, Kamal MA, Davis JD, Huniti N, Xu C, Bansal A, *et al*. Base and covariate population pharmacokinetic analyses of dupilumab in adolescents and children ≥ 6 to < 12 years of age using phase 3 data. Clin Pharmacol Drug Dev. 2021;10(11):1345–57.
